# Cavitation-enhanced carbonation for nano-ZnO synthesis via an ultrasonic–jet coupled reactor: Machine learning prediction and multi-objective optimization using a genetic algorithm

**DOI:** 10.1016/j.ultsonch.2026.107859

**Published:** 2026-04-17

**Authors:** Jinyuan Guo, Honglei Yu, Dexi Wang, Lin Fan

**Affiliations:** School of Mechanical Engineering, Shenyang University of Technology, 111, Shenliao West Road, Shenyang 110870, China

**Keywords:** Ultrasonic-jet, Cavitation intensification, Nano-ZnO, Machine learning, Sonochemistry

## Abstract

Nano-sized zinc oxide (Nano-ZnO) is of significant interest in catalysis, adsorption, rubber reinforcement, and electronic devices due to its high specific surface area, tunable crystal structure, and superior interfacial properties. However, the traditional carbonization process is often constrained by inefficient gas–liquid-solid mass transfer, the formation of a dense “self-passivation layer” on particle surfaces, and severe agglomeration during crystal growth. Consequently, achieving a synergistic enhancement in reaction yield, specific surface area, and crystallite size reduction remains a significant challenge. In this study, a 20 kHz clamp-mounted horn-type ultrasonic vibration unit coupled with an ultrasonic–jet cavitation reactor was developed to address these issues. Through multi-scale synergistic intensification involving macroscopic turbulent shear and microscopic cavitation effects, the reactor significantly enhances the interfacial renewal rate, mass transfer efficiency, and nucleation density, thereby modulating carbonization kinetics and improving product microstructure. Based on the Box-Behnken Design (BBD), the effects of four key operating parameters—ultrasonic axial distance, solid–liquid ratio, incident pressure, and jet outlet height—on reaction yield, BET specific surface area, and crystallite size were systematically investigated. Four machine learning models—BP-ANN, SVR, RF, and XGBoost—were constructed and evaluated using the BBD experimental dataset. Comparative analysis revealed that the XGBoost model exhibited superior predictive performance (R^2^ = 0.956), significantly outperforming the other three models. Furthermore, a multi-objective integrated optimization framework was established by coupling XGBoost with a genetic algorithm. The optimal process parameters were determined as follows: t = 90 min, T = 80°C, ultrasonic power = 700 W, ultrasonic transducer axial distance = 60.26 mm, solid–liquid ratio = 5.72:100, incident pressure = 0.756 MPa, and jet outlet height = 338.41 mm. Experimental validation demonstrated high consistency with model predictions, achieving a reaction yield of 94.92%, a specific surface area of 62.12 m^2^ g^−1^, and a crystallite size of 18.89 nm. Calorimetric measurements further showed that the net ultrasonic calorimetric power delivered to the liquid phase was 194.90 W. Based on the optimized treatment time of 90 min, the net ultrasonic energy input was estimated to be 1052.46 kJ. Mechanism analysis indicated that the synergistic ultrasonic-jet cavitation effectively disrupts the self-passivation layer, promotes efficient CO_2_ mass transfer, enhances nucleation density, and inhibits secondary agglomeration. Consequently, the synthesized product exhibits higher crystallinity, a well-developed mesoporous structure, and a narrower crystallite size distribution. The proposed machine-learning-assisted genetic algorithm optimization strategy successfully addresses the challenges associated with multivariable nonlinear coupling. This work demonstrates the potential of this strategy in complex chemical process intensification and provides a novel technical route and theoretical basis for the green, controllable, and efficient preparation of high-performance Nano-ZnO.

## 1 Introduction

Nano-sized zinc oxide (Nano-ZnO) is widely utilized in diverse fields such as rubber reinforcement [1], catalytic materials [2], adsorbents [3], antibacterial agents [4], and optoelectronic devices [5]. Owing to its high specific surface area, tunable crystal structure, and exceptional optical, electrical, catalytic, and adsorption properties. Among various synthesis strategies, wet carbonization is recognized as one of the most prevalent routes in both industrial and academic settings, characterized by its distinct advantages including low energy consumption [6], mild operating conditions [7], and high equipment versatility [8]. However, the conventional carbonization process is frequently constrained by inefficient mass transfer at the gas–liquid–solid interface, limitations associated with in-situ growth, and a high susceptibility to secondary agglomeration during the particle growth phase. These limitations inevitably lead to reaction stagnation, compromised overall yields, restricted specific surface area, and non-uniform crystallite size distribution [9]. Consequently, the exploration of novel intensification technologies capable of enhancing mass transfer across multiple scales, overcoming diffusion limitations, and precisely modulating particle nucleation–growth behavior has emerged as a pivotal research direction for the green and efficient preparation of high-performance Nano-ZnO.

Jet reactors significantly enhance mass transfer efficiency and improve mixing uniformity at the particle scale through mechanisms such as high-velocity fluid-induced turbulent shear [10], entrainment mixing [11], and rapid interfacial renewal [12]. Existing studies have confirmed the excellence of hydrodynamic cavitation technology in intensifying multiphase flow processes. For instance, Zuo et al. [13] effectively overcame the problem of in-situ crystal growth during precipitation using hydrodynamic cavitation, successfully preparing high-quality Mg(OH)_2_ with good dispersibility. Guo et al. [14] employed a hydrodynamic cavitation-assisted green synthesis route, which not only improved reaction efficiency but also yielded rod-like Nano-ZnO with fine particle size and uniform dispersion. Yang et al. [15] applied this technique to oily sludge treatment, achieving efficient separation of the oil phase (the oil content decreased from 24.9% to 9.8%, and the TPH removal rate reached 84.8%). Although hydraulic jets exhibit clear advantages in enhancing macroscopic mixing, relying solely on hydraulic shear still makes it difficult to completely eliminate the dense deposition and agglomeration of precursors on particle surfaces. This is because jet energy dissipation is mainly concentrated at the macroscopic flow-field scale, offering relatively limited capability for detachment and renewal at the microscopic solid–liquid interface. In contrast, ultrasonic cavitation—capable of generating microscale high-energy shock waves [16], transient micro-zones of extremely high temperature and pressure [17], microjets, and intense shear turbulence [18] has been demonstrated to possess unique advantages in intensifying liquid-phase micromixing, promoting interfacial renewal, suppressing agglomeration, and restructuring material microstructures [19]. Parvizian et al. [20] developed a novel sonochemical reactor based on ultrasonic cavitation and achieved synergistic optimization of both macroscopic and microscopic mixing, with an efficiency improvement of 10% compared with conventional stirred reactors. Liu et al. [21] proposed a green extraction process for vanadium slag using ultrasonic cavitation, achieving simultaneous improvements in extraction efficiency and energy consumption control. However, a single ultrasonic field in large-volume reactors suffers from severe acoustic attenuation along the propagation path, cavitation-cloud “shielding effects,” and phase decoherence, which constrain its effective action range and lead to unstable cavitation efficiency.

Coupling ultrasonic cavitation with a hydraulic jet (i.e., an Ultrasonic Jet Reactor) represents a highly promising intensification pathway [22]. The jet provides ultrasound with a greater abundance of cavitation nuclei and a highly turbulent background field, while ultrasound introduces microscale perturbations with much higher energy density to further enhance jet-induced mixing. The two fields can be spatially co-located in the throat or diffuser section, forming a synergistic mechanism of “macroscale high-speed shear and microscale cavitation fragmentation.” As a result, the CO_2_ mass-transfer rate can be significantly enhanced across multiple scales, deposition-layer detachment can be promoted, and the nucleation density can be increased [23]. Such acoustically–hydrodynamically coupled reactors open up new process opportunities for the controllable synthesis of high-quality nano-ZnO; however, their intensification mechanisms are highly complex and involve multi-variable coupling. Traditional mechanistic models have limited capability in representing such complex nonlinear behaviors, making it difficult to accurately describe the system response and meet multi-objective optimization requirements.

Machine learning (ML), owing to its strong capability for nonlinear fitting and its advantage in representing multi-factor coupling relationships, has rapidly advanced in the prediction of materials and chemical processes [24]. Among them, models such as XGBoost, SVR, RF, and BP-ANN can achieve high-accuracy prediction for strongly nonlinear processes [25]. Furthermore, integrating ML with genetic algorithms (GA) enables global search in continuous parameter spaces, providing an efficient pathway for intelligent optimization of complex industrial systems [26]. Although ML has been applied in related studies, multi-objective optimization for complex coupled systems involving “cavitation–reaction–crystallization”—which feature multiphase flow and microscopic kinetics—remains relatively limited. Therefore, this paper develops an integrated framework combining experimental investigation, response surface analysis, multi-model machine learning, and GA-based global optimization. Using this framework, we systematically investigate the multiscale intensification mechanisms and synergistic parameter regulation of nano-ZnO production via CO_2_ carbonation in an ultrasonic jet reactor. The proposed approach provides new evidence for the theoretical interpretation of ultrasonic–jet coupled intensified carbonation and offers reliable data support and optimization strategies for the green, controllable, industrial-scale synthesis of high-performance nano-ZnO.

## 2 Methods

### 2.1 Raw materials

The high-purity zinc oxide (99.5 wt% ZnO) used in this study was supplied by Liaoning Boshi Technology Co., Ltd. It is a by-product from the wet-process production of sodium hydroxymethanesulfinate, obtained by high-temperature calcination of zinc sludge. Chemical analysis indicated that the feedstock contained 99.5 wt% ZnO, while only trace impurities were detected, including PbO and CdO (0.0005 wt% each), MnO (0.001 wt%), CuO (0.002 wt%), and hydrotrope residue (0.10 wt%). Carbon dioxide was supplied in compressed-gas cylinders with a volume fraction of 98.5%, and deionized water was produced in-house.

### 2.2 Equipment and instruments

The total volume of the carbonation reactor is 40 L, and the working volume in the experiments was maintained at 50% to 60% of the reactor capacity. The main body of the reaction system was fabricated from 304 stainless steel to ensure good corrosion resistance and structural stability. The system is equipped with a self-priming flexible pump (MPR-20) with a rated adjustable power of 0.55 kW, a head of 30 m, and a recirculation flow rate of 50 L min^−1^. A variable-frequency drive (VFD) was used to regulate the pump speed, enabling precise control of the injector inlet pressure and the recirculation flow rate. The inlet pressure was maintained within 0.4 to 0.8 MPa, while the constrained pressure inside the reactor was stably controlled at 0.3 MPa. The reaction temperature was regulated by a heat-exchange system consisting of a heat exchanger, a temperature sensor, and a control unit, with a maximum operating temperature of 100°C. The control system monitored the operating conditions in real time, ensuring that the ambient temperature fluctuation throughout the reaction process remained within ±1°C. Ultrasound was supplied by a 20 kHz clamp-mounted horn-type ultrasonic vibration unit (maximum generator power: 1000 W; Hangzhou Chenrong Ultrasonic Equipment Co., Ltd., China) coupled to the pipe section of the ultrasonic–jet cavitation reactor. The ultrasonic assembly consisted of an ultrasonic generator, a vibration head, a horn, and a clamp fixture, and was used to deliver high-intensity acoustic energy directly into the jetting zone to enhance cavitation. Other testing and characterization instruments included an HCT-4 thermal analyzer, a DL-5C filtration unit, a DHG-9055A benchtop forced-air drying oven, a ZEISS Gemini 300 scanning electron microscope (SEM), a Rigaku Ultima IV X-ray diffractometer (XRD), a Malvern Mastersizer 2000 laser particle size analyzer, an S500-B multiparameter meter, and a specific surface area and pore-size analyzer supplied by Beijing Zhonghanghui Technology Co., Ltd. (Beijing, China).

**Fig. 1:**
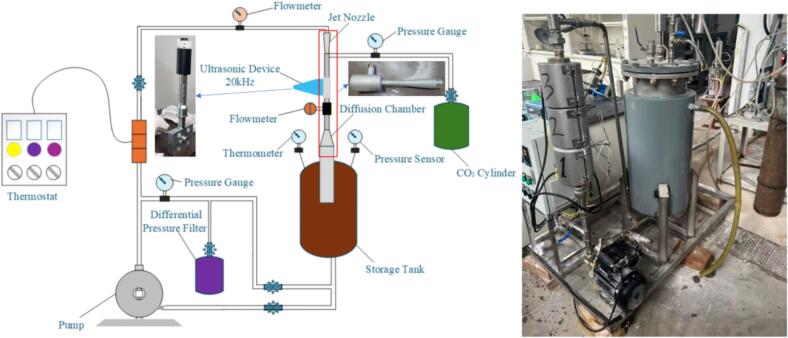
Carbonation experimental setup.

**Fig. 2:**
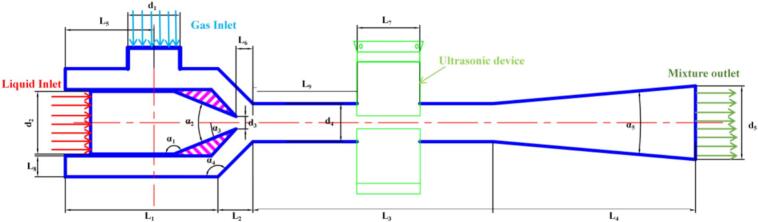
Ultrasonic–jet experimental setup.

Fig. 1 presents the overall configuration of the experimental setup for ultrasonic–jet intensified ZnO carbonation. The system mainly consists of a jacketed carbonation tank, an ultrasonic–jet cavitation reactor, a power-fluid pump, a differential-pressure filter, a heating unit, a temperature controller, a CO_2_ cylinder, and other auxiliary instruments. A schematic of the ultrasonic–jet cavitation reactor is shown in Fig. 2, and the corresponding structural parameters are listed in Table 1.

**Table 1:** Structural parameters of the ultrasonic–jet device.

Structural parameters	Size
d_1_(mm)	25.00
d_2_(mm)	30.00
d_3_(mm)	6.00
d_4_(mm)	18.00
D_5_(mm)	35.00
L_1_(mm)	76.00
L_2_(mm)	17.00
L_3_(mm)	115.00
L_4_(mm)	97.00
L_5_(mm)	42.50
L_6_(mm)	8.00
L_7_(mm)	30.00
L_8_(mm)	10.00
L_9_(mm)	50.00
*α*_1_(°)	158.00
*α*_2_(°)	45.00
*α*_3_(°)	25.00
*α*_4_(°)	135.00
*α*_5_(°)	10.00

### 2.3 Calorimetric determination of ultrasonic power

The actual ultrasonic power delivered to the liquid phase was determined by calorimetry. Independent calorimetric measurements were performed under non-reactive conditions using 10 L of deionized water as the working medium in the same circulation system as that used in the synthesis experiments. To avoid interference from external thermal inputs and to minimize heat losses, both the heating and cooling systems were switched off during the calorimetric test. The liquid temperature in the storage tank was recorded every 1 min for 10 min. Two measurements were conducted: (i) jet-only operation, with the ultrasonic generator switched off, to determine the thermal baseline associated with liquid recirculation and hydraulic dissipation; and (ii) ultrasonic–jet operation, with the remaining operating conditions unchanged, to determine the total thermal power of the coupled system.

The calorimetric power, *P*_cal_(W), was calculated according to Eq. (1):

(1) $$P_{\text{cal}}=mC_{p}\left(\frac{\mathrm{dT}}{\mathrm{dt}}\right)$$

where *m* (kg) is the mass of water, *C_p_*(J kg^−1^ K^−1^) is the specific heat capacity of water, and *dT*/*dt* (K s^−1^) is the slope of the temperature–time curve obtained by linear fitting over the initial linear interval. The net ultrasonic calorimetric power was determined by subtracting the jet-only baseline from the total calorimetric power measured under ultrasonic–jet operation, as expressed in Eq. (2):

(2) $$P_{\text{US,net}}=P_{\text{US + jet}}-P_{\text{jet}}$$

where *P*_US+jet_ is the calorimetric power under ultrasonic–jet operation and *P*_jet_ is the calorimetric power under jet-only operation. The ultrasonic energy input during treatment was estimated as:

(3) $$E_{\text{US}}=P_{\text{US,net}}t$$

where *E*_US_ is the net ultrasonic energy input and *t* is the treatment time. During the calorimetric measurements, the ultrasonic generator was operated at the same nominal power setting as that used under the optimized synthesis condition (700 W), while the remaining circulation conditions were kept unchanged. The 0 to 7 min interval was used for linear fitting to determine the initial temperature-rise rate and minimize the influence of thermal plateauing.

### 2.4 Synthesis method of ZnO nanoparticles

**Fig. 3:**
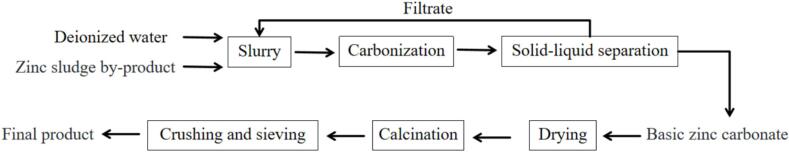
Process flow diagram for nano-ZnO synthesis.

As shown in Fig. 3, the process flowchart for nano-ZnO preparation is presented. High-purity zinc oxide (99.5 wt% ZnO) was used as the feedstock. ZnO and deionized water were charged into a jacketed carbonation tank at a prescribed solid–liquid ratio to prepare a slurry. CO_2_ was then introduced, and the power-fluid pump was started. During carbonation, the pump discharge pressure was adjusted to maintain a confined pressure of 0.3 MPa in the carbonation tank. Five aliquots were withdrawn at different times during the reaction (every 30 min) to determine the reaction yield. When the yield showed no further appreciable increase at a reaction time of 90 min, the CO_2_ supply was stopped. The system was depressurized by opening the vent valve, after which the slurry was discharged through the bottom valve. The slurry was subjected to vacuum filtration; the filtrate was either recycled for slurry preparation or discharged. The obtained filter cake was washed three times and then dried in a constant-temperature drying oven at 110°C for 4 h. Subsequently, the dried cake was calcined in a muffle furnace at 500°C for 1 h. After cooling, the calcined product was milled using a circulating-tube jet mill and sieved to remove oversized residues, yielding the final nano-ZnO product.

The reaction equations are given in Eqs. (4) to (5).

(4) 5ZnO + 3H_2_O + 2CO_2_ → Zn_5_(CO_3_)_2_(OH)_6_

(5) Zn_5_(CO_3_)_2_(OH)_6_ → 5ZnO + 3H_2_O + 2CO_2_

**Fig. 4:**
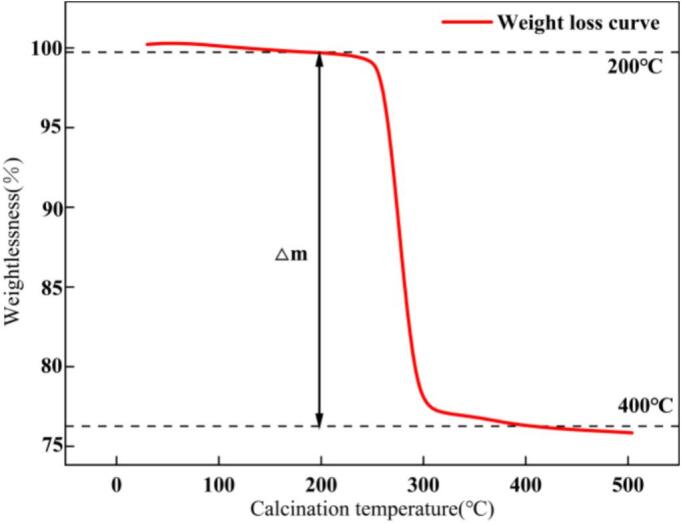
TGA curves.

As shown in Fig. 4, the decomposition steps of basic zinc carbonate—including the release of crystallization water, hydroxyl groups, and carbonate species—exhibit a high degree of overlap within the investigated temperature range. The evolution temperatures of these components are very close, and the corresponding mass-loss events occur within the same interval (200 to 400°C). As a result, under different heating rates, the overall decomposition behavior appears as a single, continuous mass-loss region. Therefore, a small amount of the dried basic zinc carbonate sample obtained after carbonation was weighed to calculate the theoretical masses of CO_2_ and H_2_O. Thermogravimetric analysis (TGA) was then performed to measure the total mass of CO_2_ and H_2_O released during the thermal decomposition of basic zinc carbonate. The ZnO reaction yield was subsequently calculated using Eq. (6) (the average value was determined from three replicate measurements).

(6) $$\Phi =\left[\frac{m_{2}}{m_{1}}\right]\times 100\%$$

where Φ denotes the ZnO reaction yield, m_1_ is the theoretical mass of CO_2_ and H_2_O, and m_2_ is the actual mass of CO_2_ and H_2_O measured by thermogravimetric analysis (TGA).

**Fig. 5:**
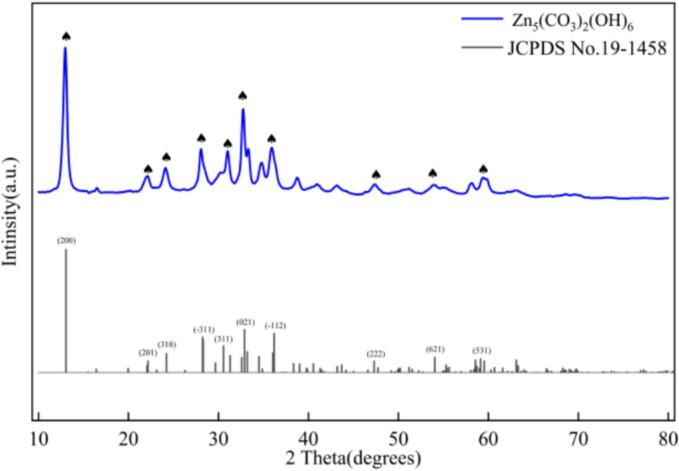
XRD pattern of Zn_5_(CO_3_)_2_(OH)_6._

Fig. 5 shows the XRD pattern of the carbonated product, confirming that the nano-ZnO precursor formed after carbonation is Zn_5_(CO_3_)_2_(OH)_6_.

### 2.5 Box–Behnken design (BBD)

In this study, a Box–Behnken design (BBD) with four independent variables was employed. A total of 27 experimental runs were conducted using Design-Expert 13, and the complete design matrix together with the corresponding response values is provided in Table S1 of the Supplementary Material. The four investigated factors were ultrasonic axial distance (A), solid–liquid ratio (B), inlet pressure (C), and jet outlet height (D). Each factor was coded at three levels (−1, 0, and 1). Three key quality attributes—reaction yield, BET specific surface area, and crystallite size—were selected as the responses. The reaction yield was determined according to the method described in Section 2.4. The specific surface area of nano-ZnO was measured using a surface area and pore-size analyzer. The crystallite size was characterized by the XRD line-broadening method in accordance with GB/T 19589–2004: the full width at half maximum (FWHM) of the characteristic diffraction peak was measured, and the crystallite size was calculated using the Scherrer equation. On this basis, a second-order polynomial model was used to fit the experimental data and establish the regression relationships between the response variables and the process parameters.

Y = X_0_ + X_1_A + X_2_B + X_3_C + X_4_D + X_5_A^2^ + X_6_B^2^ + X_7_C^2^ + X_8_D^2^ + X_9_AB + X_10_AC + X_11_AD + X_12_BC + X_13_BD + X_14_CD

where (Y) is the response variable; A, B, C, and D are the four independent factors; and X_1_, X_2_, X_3_, and X_4_ are the linear regression coefficients. The quadratic terms are represented by X_5_, X_6_, X_7_, and X_8_, while X_9_, X_10_, X_11_, X_12_, X_13_, and X_14_ denote the interaction coefficients of the four factors.

### 2.6 Machine-learning models

Unlike conventional response surface methodology (RSM), which presumes a quadratic relationship, machine-learning (ML) models were introduced to capture the highly nonlinear interactions induced by cavitation–jet coupling. The experimental factors included A: ultrasonic axial distance, B: solid–liquid ratio, C: inlet pressure, and D: jet outlet height; these four variables were used as the model inputs. Three key quality attributes of the nano-ZnO product—reaction yield, BET specific surface area, and crystallite size—were selected as the output responses. In the ML modeling stage, four algorithms were implemented in a Python 3.12 environment on the Jupyter Notebook platform, namely a back-propagation artificial neural network (BP-ANN), extreme gradient boosting (XGBoost), random forest (RF), and support vector regression (SVR). The dataset was obtained from 27 experimental runs, each conducted in triplicate, yielding 81 valid data points. All data were split into a training set and a test set using random stratified sampling: 85% of the samples were used for model training (n = 68) and 15% for model testing (n = 13). The split was performed to ensure that the training set covered the full variation ranges of both the input variables and the output responses, while the test set was randomly drawn from the remaining samples and constrained to lie within the training-set parameter space to avoid extrapolation-induced errors. To enhance model robustness, 10-fold cross-validation was performed for all models, and model hyperparameters were systematically optimized using grid search. The primary tuning ranges included the learning rate (1 × 10^-4^ to 1 × 10^-3^), the number of trees (RF and XGBoost: 50 to 200), and the kernel coefficient (SVR: 0.01 to 1.0). During cross-validation, minimization of the mean squared error (MSE) was adopted as the criterion for selecting the optimal model.

The predictive performance of the models was comprehensively evaluated using the coefficient of determination (R^2^), mean squared error (MSE), root mean squared error (RMSE), and mean absolute error (MAE). The corresponding equations are given as follows:

(7) $$R^{2}=1-\frac{\sum_{i=1}^{n}\left(Y_{e}-Y_{p}\right)}{\sum_{i=1}^{n}\left(Y_{m}-Y_{p}\right)}$$

(8) $$\text{MSE}=\frac{\sum_{i=1}^{n}{\left(Y_{e}-Y_{p}\right)}^{2}}{n}$$

(9) $$\text{RMSE}=\sqrt{\frac{\sum_{i=1}^{n}{\left(Y_{e}-Y_{p}\right)}^{2}}{n}}$$

(10) $$\text{MAE}=\frac{\sum_{i=1}^{n}\left|Y_{e}-Y_{p}\right|}{n}$$

where Y_e_ denotes the experimental value, Y_p_ the predicted value, Y_m_ the mean value, and n the total number of experiments. Although the experimental dataset in this study is relatively limited (n = 81), adopting a 10-fold cross-validation strategy maximizes data utilization, enables an effective assessment of model generalization, and helps prevent overfitting. This strategy has been widely demonstrated to be reliable and necessary in ML-assisted studies of chemical processes with small sample sizes.

#### 2.6.1 BP-ANN

BP-ANN is one of the most widely used ML algorithms and has been extensively applied to regression and classification tasks across diverse fields [27]. In this study, a BP-ANN model was employed to estimate three key quality attributes in the nano-ZnO preparation process using the ultrasonic–jet reactor. The network topology was configured with two hidden layers, each containing six neurons with the ReLU activation function. Compared with a single-hidden-layer architecture, the two-hidden-layer network can more efficiently capture complex nonlinear relationships among the input variables (e.g., the coupling between the acoustic and flow fields) through hierarchical feature extraction. Meanwhile, this compact architecture keeps the number of trainable parameters within a reasonable range and, when combined with a rigorous 10-fold cross-validation strategy, effectively balances fitting capability and generalization performance, thereby reducing the risk of overfitting under the small-sample condition (n = 81). Model training was performed using the Adam optimizer with a learning rate of 5 × 10^-4^.

#### 2.6.2 SVR

Support vector regression (SVR) is an algorithm grounded in the statistical learning principle of structural risk minimization and is widely used for solving nonlinear regression problems [28]. SVR can be implemented with different kernel functions, resulting in various SVR formulations. Owing to its strong generalization capability and robustness, SVR is well suited for modeling complex nonlinear systems. In this study, the SVR model was implemented in Python 3.12 using the scikit-learn library, with the radial basis function (RBF) adopted as the kernel. Hyperparameters were optimized via grid search, and the kernel coefficient (γ) was set to 0.1, while the regularization parameter (C) was set to 100 to achieve a balanced trade-off between model bias and variance.

#### 2.6.3 RF

Random forest (RF) performs classification or regression by constructing an ensemble of decision trees with injected randomness, thereby improving predictive accuracy and generalization ability [29]. In this study, the RF model was implemented in Python 3.12 using the scikit-learn library. The model comprised 100 decision trees (n_estimators = 100) and employed bootstrap resampling to enhance sample diversity. No maximum tree depth was imposed, allowing the trees to grow fully so as to capture subtle patterns in the data.

#### 2.6.4 XGBoost

XGBoost is an efficient algorithmic toolkit based on the boosting framework, exhibiting strong performance in parallel computation, missing-value handling, and predictive accuracy [30]. In this study, XGBoost was implemented in Python 3.12 using the XGBoost library. The model was configured with 150 gradient-boosted trees, a maximum tree depth of 4, and a learning rate of 0.1.

### 2.7 Genetic algorithm-based optimization

A genetic algorithm (GA) was embedded in the Jupyter Notebook platform using Python 3.12 to optimize the process parameters (Fig. 1B). The established machine-learning models were coupled with the GA to search the input parameter space and identify the optimal combination that yields the best output quality attributes. The GA settings were as follows: a population size of 1000 and 100 generations. Both the crossover and mutation probabilities were set to 0.5, and tournament selection was adopted with a tournament size of 3 [31]. The fitness function was formulated to maximize BET specific surface area and reaction yield while minimizing crystallite size, with equal weights assigned to the three key quality attributes. The optimal parameter set was determined by maximizing the fitness value.

## 3 Results and discussion

### 3.1 Single-factor experimental analysis

(1) Reaction time

**Fig. 6:**
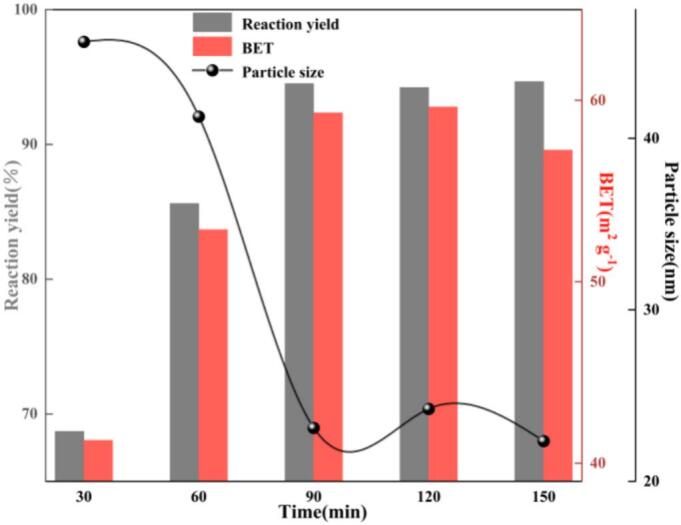
Effect of reaction time on reaction yield, BET specific surface area, and crystallite size.

As shown in Fig. 6, as the residence time (t) increased from 30 to 90 min, the reaction yield (Φ) and BET specific surface area (BET) increased monotonically, whereas the average crystallite size (D) continuously decreased, exhibiting a typical “cavitation intensification with diminishing marginal returns” behavior. Mechanistically, transient cavitation in the ultrasonic–jet field continuously generates microjets and microturbulence, which markedly increase the interfacial renewal frequency and shear intensity, thin the diffusion boundary layer, and enhance the volumetric mass-transfer coefficient(*k_L_a*). Meanwhile, cavitation impacts and microjets repeatedly fragment particle agglomerates, effectively suppressing early-stage agglomeration and secondary growth, thereby promoting sustained particle refinement during the reaction and nucleation stages. At t = 90 min, the system approached its optimum performance, with Φ = 94.52%, BET = 59.31 m^2^ g^−1^, and D = 23.11 nm. When the reaction time was further extended to 150 min, both Φ and BET essentially plateaued and the variation in D became marginal, indicating that the net conversion between reactants and products tended toward a quasi-steady state and the marginal contribution of cavitation-induced mass transfer and fragmentation decreased substantially. In parallel, kinetic limitations became more pronounced, while particle re-agglomeration and Ostwald ripening intensified, partially offsetting the benefits of cavitation-driven refinement and resulting in nearly unchanged macroscopic performance. Therefore, considering the trade-off between performance enhancement and energy consumption, the optimal reaction time was determined to be t = 90 min.

(2) Reaction temperature

**Fig. 7:**
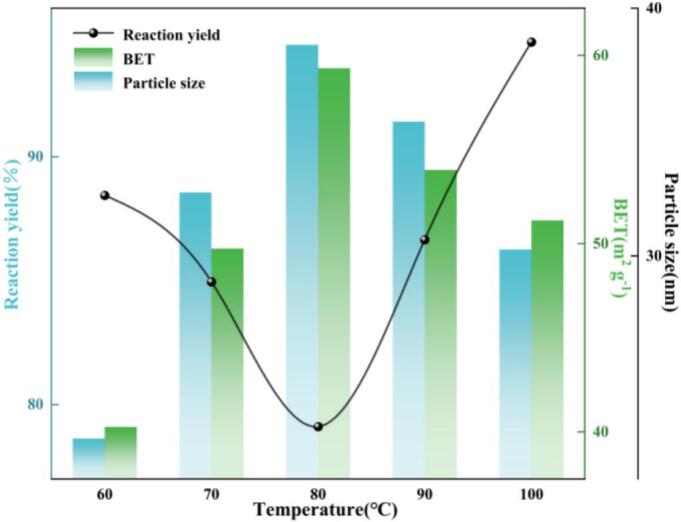
Effect of reaction temperature on reaction yield, BET specific surface area, and crystallite size.

As shown in Fig. 7, when the reaction temperature increased from 60 to 100°C, the reaction yield and BET specific surface area of nano-ZnO exhibited a non-monotonic trend—first increasing and then decreasing—whereas the crystallite size showed the opposite trend, i.e., first decreasing and then increasing. The overall optimum was obtained at 80°C (yield = 94.52%, BET = 59.31 m^2^ g^−1^, crystallite size = 23.11 nm). This behavior arises from the coupled influence of temperature on cavitation intensity, mass transfer, nucleation–growth kinetics, and particle evolution. On the one hand, a moderate temperature increase reduces liquid viscosity and surface tension while increasing the saturated vapor pressure, thereby lowering the cavitation number. Consequently, the frequency of bubble inception–collapse events and microjet formation increases, enhancing interfacial renewal and shear intensity. These effects simultaneously increase the liquid-film mass-transfer coefficient and the interfacial area per unit volume, leading to a maximum volumetric mass-transfer coefficient *k_L_a* at 80°C. In addition, the ultrasonic–jet reactor intensifies CO_2_ micromixing toward the liquid/solid interface and increases the effective supersaturation, making the nucleation rate dominant over crystal growth; cavitation impacts also suppress early-stage agglomeration. As a result, yield and BET increase while crystallite size decreases in the 60 to 80°C range. When the temperature is further increased (80 to 100°C), the equilibrium solubility of CO_2_ in the liquid phase decreases with increasing temperature (i.e., the Henry’s constant increases), which weakens the bulk supersaturation and the mass-transfer driving force(C*-*C_L_*). Meanwhile, at elevated temperatures, cavitation clouds enhance acoustic attenuation (via multiple scattering and absorption) and strengthen the vapor-cushioning effect. Hydrodynamic interference induced by interactions among neighboring bubbles reduces the pressure differential upon single-bubble collapse and the kinetic energy of microjets, thereby limiting the effective cavitation zone and the attainable *k_L_a*. Moreover, higher temperatures accelerate surface diffusion and dissolution–reprecipitation, promoting Ostwald ripening and re-agglomeration [32]. Collectively, these factors lead to a decline in BET and reaction yield, accompanied by an increase in average crystallite size. Therefore, 80°C represents an optimal balance between cavitation-assisted mass-transfer enhancement, accelerated reaction kinetics, and late-stage ripening/agglomeration, yielding simultaneously high conversion and specific surface area together with refined crystallite size—an intensification optimum of the ultrasonic–jet carbonation process along the temperature dimension.

(3) Ultrasonic power

**Fig. 8:**
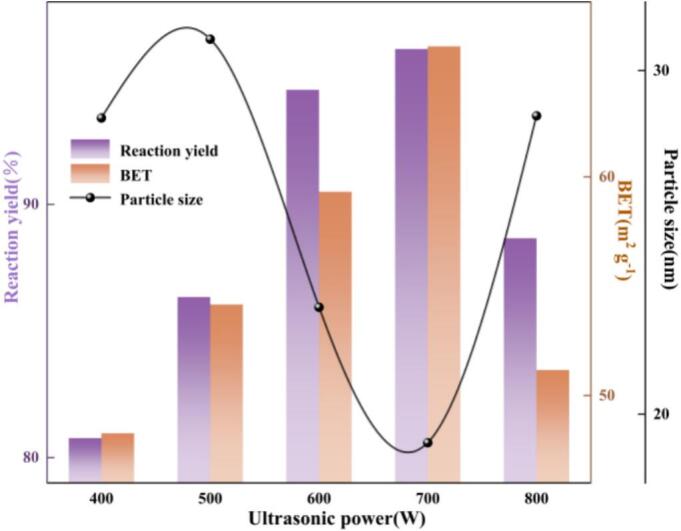
Effect of ultrasonic power on reaction yield, BET specific surface area, and crystallite size.

As shown in Fig. 8, when the ultrasonic power increased from 400 to 800 W, the reaction yield and BET specific surface area first increased and then decreased, whereas the crystallite size exhibited the opposite trend (first decreasing and then increasing). This indicates that cavitation-driven interfacial and near-surface processes were progressively intensified with increasing power. A higher acoustic pressure amplitude activates more cavitation nuclei and increases the frequency of inertial-collapse events. The resulting bubble microjets and localized turbulence enhance the gas–liquid interfacial area and renewal rate, thin the diffusion boundary layer, and increase the volumetric mass-transfer coefficient *k_L_a*, thereby accelerating CO_2_ transport into the liquid phase and toward the particle near-surface region. At the solid scale, cavitation-induced high-gradient shear and impact forces disrupt soft agglomerates, shorten mass-transfer pathways, and generate higher and more uniform supersaturation at particle surfaces and agglomerate boundaries. This promotes preferential nucleation and fine-scale deposition of basic zinc carbonate on particle surfaces, ultimately yielding a desirable combination of high yield, high BET, and small crystallite size at 700 W. When the power was further increased to 800 W, intensified bubble–bubble interactions and elevated local vapor content reduced the effective acoustic pressure and the collapse intensity of individual bubbles (i.e., cloud-cavitation decoherence and “vapor cushioning”) [33]. Meanwhile, microscale thermal effects and surface diffusion were accelerated, and dissolution–reprecipitation cycling together with late-stage ripening/re-agglomeration became more pronounced [34]. Consequently, both BET and yield decreased (to 51.16 m^2^ g^−1^ and 88.66%, respectively), while the crystallite size increased (to 28.68 nm). Therefore, ∼700 W represents the optimal power level for process intensification, maximizing CO_2_ supply, interfacial renewal, and surface deposition rate while keeping agglomeration and ripening at a relatively low level.

(4) Ultrasonic distance(L_9_)

**Fig. 9:**
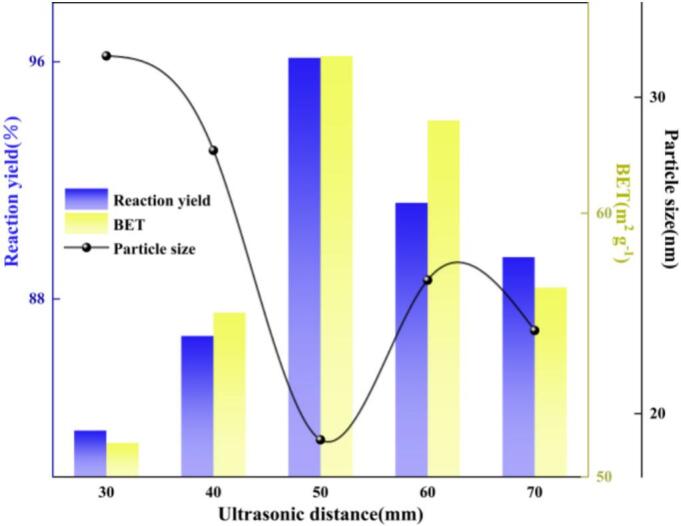
Effect of ultrasonic transducer axial distance (L_9_) on reaction yield, BET specific surface area, and crystallite size.

As shown in Fig. 9, adjusting the axial position of the transducer (30 to 70 mm) can markedly alter the macroscopic performance of nano-ZnO. At L_9_ = 50 mm, the reaction yield (96.13%) and BET specific surface area (65.96 m^2^ g^−1^) reached their maxima, while the crystallite size was minimized(19.17 nm); deviations from this position led to performance deterioration. This behavior primarily arises from the variation in spatial co-localization between the ultrasonic cavitation intensity field and the jet-induced gas–liquid interfacial renewal zone as (L_9_) changes, which in turn governs the accessible interfacial renewal and the efficiency of selective fragmentation. Let the axial spectrum of inertial cavitation intensity be denoted as *I*_cav_(*x*;*L*_9_), and the shear-weighting function associated with the nozzle contraction–throat–downstream diffuser region be represented by *χ*_mix_(*x*). Then, the “effective cavitation dose” [35] can be formally expressed as:

(11) $$\Psi \left(L_{9}\right)=\int I_{\mathrm{cav}}\left(x;L_{9}\right)\chi_{\mathrm{mix}}\left(x\right)dx$$

When L_9_≈50 mm, the acoustic “hot spot” overlaps with the active zone near particle surfaces, leading to a simultaneous increase in the inertial cavitation event frequency and the interfacial renewal rate per unit volume, thereby elevating the effective volumetric mass-transfer coefficient *k_L_a*. At the particle scale, cavitation microjets and shock waves not only enhance the local supply of CO_2_ and refresh the diffusion boundary layer, but also selectively fragment soft agglomerates, promoting preferential nucleation and deposition of basic zinc carbonate on the ZnO surface. Consequently, a synergistic improvement of “high yield, high BET, and small crystallite size” is achieved [36]. When L_9_ < 50 mm, the acoustic hot spot is located too close to the contraction section and the wall. Coupling between cavitation bubbles and the wall induces non-spherical collapse, causing energy dissipation, limiting the effective active volume, and increasing phase decoherence, which reduces Ψ(*L*_9_). When L_9_ > 50 mm, the incident acoustic pressure attenuates along the propagation path and bubble clouds are more prone to enter a decoherent cloud-cavitation regime. The collapse intensity and synchronicity of individual bubbles decrease, resulting in partial decoupling between cavitation and mass transfer; consequently, both *k_L_a* and the selective fragmentation efficiency decline. Overall, L_9_ = 50 mm was selected as the ultrasonic axial distance for subsequent experiments.

(5) Solid-liquid ratio

**Fig. 10:**
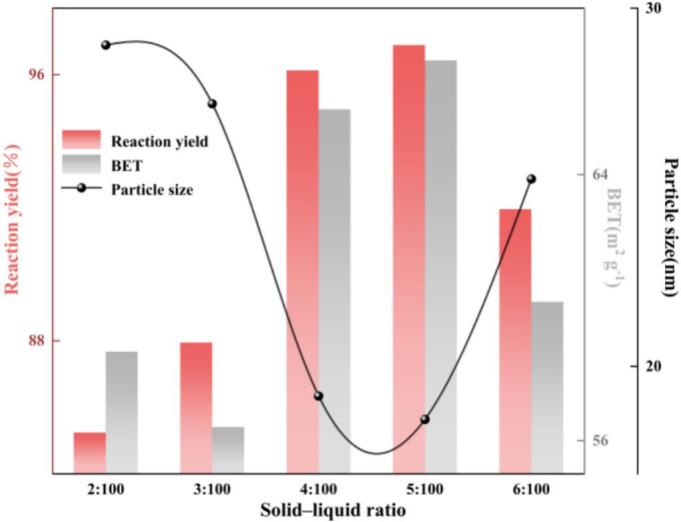
Effect of solid-to-liquid ratio (S/L) on reaction yield, BET specific surface area, and crystallite size.

As shown in Fig. 10, the solid-to-liquid ratio (S/L) exhibits a unimodal intensification effect on the overall reaction system. At an S/L of 5:100, both the reaction yield and BET specific surface area reached their maximum values (96.89% and 67.43 m^2^ g^−1^, respectively), while the crystallite size was minimized (18.52 nm). Compared with an S/L of 2:100, the yield increased by 11.65%, BET increased by 8.76 m^2^ g^−1^, and the crystallite size decreased by 10.45 nm. In contrast, when S/L was increased from 5:100 to 6:100, the yield and BET decreased by 4.93% and 7.26 m^2^ g^−1^, respectively, and the crystallite size increased by 6.71 nm. At the dilute end (2:100), the solid volume fraction and effective surface area are insufficient, resulting in a low density of heterogeneous nucleation sites. In addition, the acoustically induced microstreaming and jet-induced shear field near particles are relatively weak. Although such conditions facilitate CO_2_ dissolution into the liquid phase, the generation and maintenance of near-surface supersaturation are inadequate, causing deposition to be biased toward crystal growth rather than nucleation; consequently, lower yield and BET, together with larger crystallite size, are observed. As S/L increases to 5:100, the solid phase provides abundant and more uniformly distributed heterogeneous surfaces, while the slurry viscosity remains insufficient to significantly suppress cavitation. The frequency of inertial-collapse events and the interfacial renewal rate increase, the diffusion boundary layer becomes thinner, and the volumetric mass-transfer coefficient (*k_L_a)* improves. Enhanced CO_2_ supply and accelerated near-surface species rearrangement render nucleation dominant, accompanied by ultrasonically induced selective deagglomeration, thereby achieving a synergistic optimum of “high yield–high BET–small crystallite size.” When S/L is further increased to 6:100, the higher solids loading and slurry viscosity intensify acoustic attenuation and scattering. Interactions between cavitation bubbles and particles become stronger, favoring the formation of decoherent cloud cavitation, which reduces the collapse intensity and synchronicity of individual bubbles. Moreover, the high S/L increases local Zn^2+^ supersaturation, triggering dense secondary nucleation and surface re-deposition; part of the deposited layer may even encapsulate unreacted ZnO via in-situ growth. As a result, both yield and BET decline and the crystallite size increases. Overall, an S/L of 5:100 was selected for subsequent experiments.

(6) Incident pressure

**Fig. 11:**
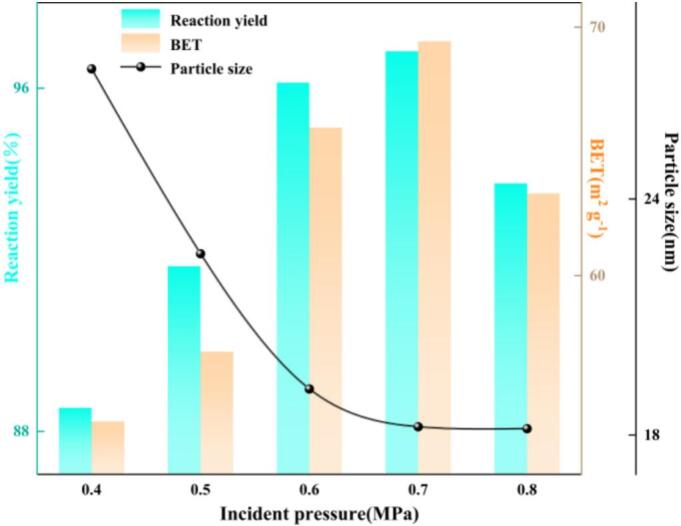
Effect of incident pressure on reaction yield, BET specific surface area, and crystallite size.

As shown in Fig. 11, when the slurry incident pressure increased from 0.4 to 0.8 MPa, the system exhibited a non-monotonic response characterized by “intensification followed by saturation”. Increasing the pressure from 0.4 to 0.6 MPa led to a pronounced rise in reaction yield and BET specific surface area, accompanied by a rapid decrease in crystallite size. Further increasing the pressure from 0.6 to 0.7 MPa still provided additional benefits, whereas at 0.8 MPa a marginal deterioration was observed and the crystallite size became nearly unchanged. These results indicate that inlet pressure strongly affects the cavitation number (*σ*).The cavitation number describes the relationship between the difference of the local absolute pressure and the saturated vapor pressure of the liquid and the local kinetic energy per unit volume, and is a dimensionless parameter commonly used to characterize the likelihood of cavitation inception in a flowing liquid. Its expression is given in Eq. (12): a lower *σ* corresponds to a higher cavitation tendency, whereas a higher *σ* indicates that cavitation is unlikely to occur [37].

(12) $$\sigma =\frac{P-P_{\nu }}{\frac{1}{2}\rho u^{2}}$$

where *ρ* denotes the liquid density; *P* is the local absolute pressure; *P_v_* is the saturated vapor pressure of the liquid at the ambient temperature; and *u* is the liquid flow velocity.

Increasing the inlet pressure raises the flow velocity in the reactor throat (*u*) and consequently reduces the cavitation number (*σ*). Under such conditions, a larger fraction of metastable cavitation nuclei can surpass the Blake threshold and approach their resonance radius, leading to an increased frequency of inertial cavitation events per unit volume. With improved spatial co-localization between cavitation activity and the jet shear layer, the interfacial renewal rate and microscale shear intensity are simultaneously enhanced, markedly thinning the diffusion boundary layer and increasing the effective volumetric mass-transfer coefficient (*k*_L_*a*). At the particle scale, nucleation becomes more competitive relative to crystal growth, while selective deagglomeration of early-stage soft agglomerates is promoted, thereby sustaining the carbonation process. When the inlet pressure is further increased to 0.8 MPa, although *σ* decreases further, the jet core becomes excessively extended and the void fraction increases, promoting a transition from discrete bubbles to a cloud-cavitation regime. Bubble–bubble interactions then intensify phase decoherence and vapor cushioning, such that the effective pressure differential during single-bubble collapse and the kinetic energy of microjets no longer increase. Overall, an inlet pressure of 0.6 MPa was selected for subsequent experiments.

(7) Different jet positions(H)

**Fig. 12:**
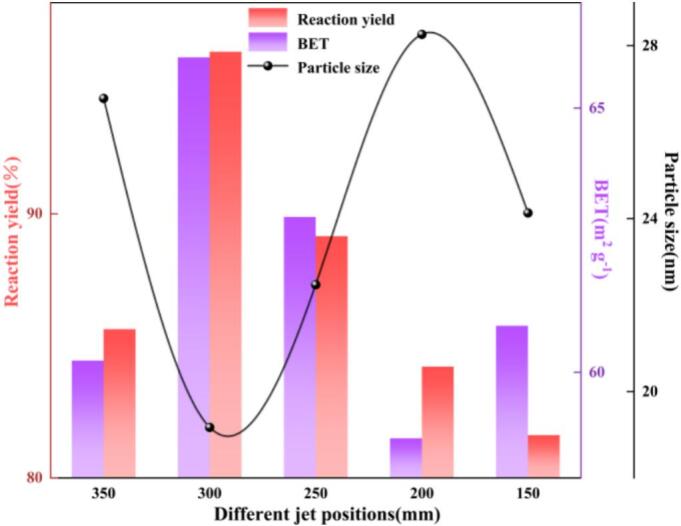
Effect of different jet positions on reaction yield, BET specific surface area, and crystallite size.

As shown in Fig. 12, varying the jet position in the reactor—quantified by the axial height (H) (defined as the distance from the reactor top to the jet outlet)—significantly affects the reaction yield, BET specific surface area, and crystallite size of nano-ZnO. As H decreased from 350 to 300 mm, the reaction yield and BET increased continuously, while the crystallite size decreased. The optimum performance was obtained at H = 300 mm (yield = 96.13%, BET = 65.96 m^2^ g^−1^, crystallite size = 19.17 nm).At H = 300 mm, the hydrodynamic cavitation zone and the jet shear layer establish a stable recirculating annular-flow structure within the main body of the reactor. The jet potential core has not yet significantly decayed, and sufficient clearance from both the vessel wall and the free liquid surface is maintained. These conditions sustain high shear and high interfacial renewal frequency over a relatively large effective volume, thereby increasing the effective volumetric mass-transfer coefficient *k_L_a*. At the particle scale, nucleation becomes dominant and selective deagglomeration is promoted. When H is further increased or decreased, performance deteriorates. If H is too small (i.e., the jet outlet is too close to the reactor top), interactions with the upper boundary intensify; the potential core is reflected and laterally diverted before fully developing, which restricts the effective active volume and reduces the spatial coupling between the cavitation-intensity field and the mixing zone. Conversely, if H is too large (i.e., the jet outlet moves downward toward the reactor bottom), the jet suffers substantial momentum loss along the path, the bottom recirculation zone expands, and low-velocity stagnation and localized deposition become more likely. In addition, the coherence of the cavitation cloud decreases, making it difficult to maintain high *k_L_a* and strong microscale shear. Therefore, a jet position of H = 300 mm was selected for subsequent experiments.

### 3.2 Mechanism of nano-ZnO preparation in an ultrasonic–jet reactor

**Fig. 13:**
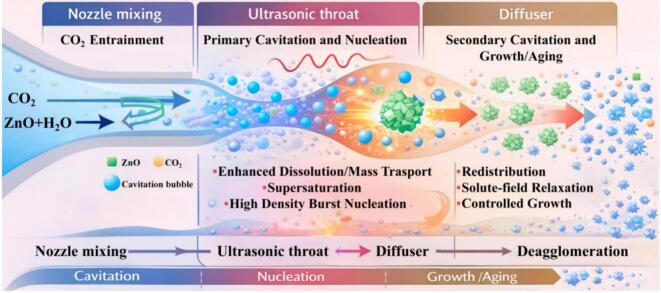
Schematic illustration of the multiphase reaction mechanism in the ultrasonic–jet reactor.

Fig. 13 schematically illustrates the multiphase reaction mechanism in the ultrasonic–jet reactor. In this reactor, the reaction of the ZnO–H_2_O–CO_2_ system is not a simple dissolution–precipitation process; rather, under the hydrodynamic–acoustic coupling induced by the specific reactor geometry, it proceeds as a cavitation-dominated, multiscale transformation. First, the high-velocity jet is accelerated through the converging nozzle and enters the ultrasonic action zone within the throat. CO_2_ is efficiently entrained by the locally reduced pressure and comes into intimate contact with the ZnO suspension. The periodic compression and rarefaction of the acoustic field promotes the inception, growth, and transient collapse of a large number of cavitation bubbles. The localized high-temperature/high-pressure microenvironments and microjets generated upon bubble collapse markedly intensify the dissolution kinetics at the ZnO particle surface, accelerating the microscale enrichment of Zn^2+^, OH^–^, and CO_3_^2–^. Under high supersaturation, dense nucleation is triggered, while persistent high-shear perturbations effectively suppress agglomeration and uncontrolled growth. Consequently, the controlling mechanism shifts from diffusion-limited behavior toward nucleation-dominated kinetics, favoring the formation of structurally stable Zn_5_(OH)_6_(CO_3_)_2_ nuclei. Subsequently, the fluid enters the diffuser section, where the flow velocity decreases and pressure fluctuations together with recirculation structures develop. In this region, secondary weak cavitation and turbulent mixing further promote the redistribution of unreacted species and the re-equilibration of the solute field surrounding the crystals. This facilitates uniform secondary growth and structural rearrangement of the primary particles, while mitigating non-uniform expansion driven by local concentration gradients. The diffuser thus constitutes a “stabilized growth stage after nucleation intensification,” maintaining effective mixing while alleviating excessively intense shear, enabling controlled size enlargement and morphology optimization without compromising particle uniformity. Overall, the reactor achieves a three-stage synergistic intensification mechanism—“nozzle-accelerated mixing, ultrasonic-enhanced nucleation in the throat, and growth regulation in the diffuser.” This integrated structural design not only markedly improves the formation efficiency of basic zinc carbonate, but also enables continuous and controllable regulation from microscopic nucleation to macroscopic crystallite-size evolution, providing a systematic structural advantage for producing nano-ZnO with high specific surface area, narrow crystallite-size distribution, and well-defined morphology.

**Fig. 14:**
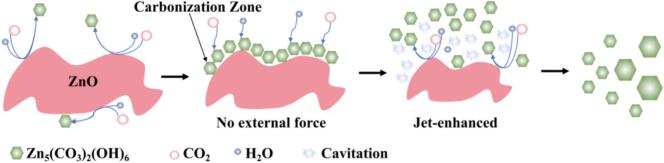
Schematic illustration of the cavitation-induced intensification mechanism for the carbonation process.

The cavitation effect significantly intensifies the carbonation of ZnO, and the underlying mechanism is illustrated in Fig. 14. Under conventional conditions, carbonation occurs as ZnO particle surfaces contact water and CO_2_, gradually forming Zn_5_(OH)_6_(CO_3_)_2_. However, because freshly formed basic zinc carbonate crystallites exhibit high surface polarity and strong interfacial adsorption, they tend to grow in situ on the ZnO surface and rapidly agglomerate, forming a dense passivation layer. This compact coating impedes further contact between unreacted ZnO and the reaction medium, resulting in mass-transfer limitations. Consequently, the reaction progressively shifts from chemical-control to diffusion-control, and the carbonation rate decreases markedly. This “self-passivation” effect is one of the fundamental reasons for the low carbonation extent, reaction stagnation, and limited specific surface area in the conventional synthesis of basic zinc carbonate using ZnO as the feedstock. With cavitation intensification, a large number of cavitation bubbles are generated in locally low-pressure regions. Their periodic growth and transient collapse release high-intensity microjets, shock waves, and localized high-temperature/high-pressure microdomains, which impose continuous mechanical perturbations on ZnO particle surfaces. These effects effectively strip off the previously formed and adhered basic zinc carbonate layer, re-exposing the passivated ZnO active surface to the reactive environment. As a result, the interfacial contact with H_2_O and CO_2_ is restored and the interfacial renewal rate is significantly increased. Meanwhile, cavitation-induced strong shear and microscale turbulence continuously fragment agglomerates and promote solute redistribution, enabling the reaction system to maintain high effective mass-transfer efficiency and supersaturation, thereby sustaining the carbonation process. This dynamic cycle of stripping–re-exposure–re-reaction transforms ZnO carbonation from a conventional “surface-sealed reaction” into a “continuously renewed interfacial reaction,” substantially accelerating the carbonation kinetics and improving the overall reaction efficiency. In addition, cavitation plays an important role in regulating the dispersion of the formed basic zinc carbonate. By weakening interparticle van der Waals interactions and electrostatic adsorption, cavitation suppresses secondary agglomeration, allowing product particles to remain highly dispersed with a more open structural morphology. This is beneficial for constructing a porous basic zinc carbonate system with high specific surface area.

### 3.3 RSM analysis

Based on the Box–Behnken design (BBD), the experimental responses for reaction yield (Y_1_), BET specific surface area (Y_2_), and crystallite size (Y_3_) were obtained, and the complete experimental matrix is presented in Table S1 of the Supplementary Material. Building on the preliminary single-factor experiments, response surface methodology (RSM) was further employed to optimize the four key parameters in the ultrasonic–jet reactor coupled carbonation process for nano-ZnO preparation, namely Ultrasonic distance (A), Solid–liquid ratio (B), Incident pressure (C), and Different jet positions (D). Analysis of variance (ANOVA) and regression analysis were conducted to evaluate the effects of these factors. Subsequently, multivariate regression was performed to fit the experimental data, and the resulting model equations are presented as follows:

Y_1_ = -179.38708 + 1.82150A + 2130.33333B + 167.67500C + 0.776133D + 7.42500AB + 1.23000AC-0.001685AD + 1325.00000BC-2.57000BD + 0.052500CD-0.024487A^2^-27000.00000B^2^-224.75000C^2^-0.000984D^2^

Y_2_ = -137.16917 + 1.88042A + 2158.25000B + 198.85833C + 0.219600D + 4.45000AB + 0.190000AC + 0.002070AD + 55.00000BC + 0.215000BD + 0.070000CD-0.028679A^2^-25616.66667B^2^-164.04167C^2^-0.000636D^2^

Y_3_=+346.74667–1.38775A-3451.83333B-322.56667C-0.638200D + 2.27500AB + 0.512500AC + 0.000495AD + 105.00000BC-0.180000BD-0.001000CD + 0.008167A^2^ + 33691.66667B^2^ + 210.16667C^2^ + 0.001033D^2^

The ANOVA results of the four-factor quadratic models for the three responses are summarized in Table S2 of the Supplementary Material. For all three responses, the overall model terms are statistically significant (p < 0.05), indicating that the quadratic polynomial models can satisfactorily explain the major variation in the experimental responses and are therefore suitable for subsequent response-surface analysis and optimization. Regarding nonlinear contributions, the quadratic terms (A^2^, B^2^, C^2^, D^2^) are all significant and non-zero, demonstrating that the effects of the factors on reaction yield, BET specific surface area, and crystallite size are not purely linear. Several interaction terms are also significant, suggesting the presence of synergistic or antagonistic effects among factors. The coefficients of determination (R^2^) are 0.8764, 0.8648, and 0.8489, respectively, with an average (R^2^) of 0.8634, indicating good goodness-of-fit. The residual standard deviations (RMSE) are 1.13, 0.9819, and 1.15, and the corresponding relative errors (normalized by the response means) fall within a low-to-moderate range, supporting satisfactory predictive accuracy. Therefore, within the investigated domain, the quadratic models can be regarded as reliable approximations for capturing process trends and conducting sensitivity analysis. It should be noted that classical response surface methodology (RSM), which relies on quadratic polynomials, is inherently constrained by the assumed functional order and additivity. On the one hand, strong nonlinearities or threshold effects may be underfitted by a quadratic form; on the other hand, RSM typically identifies a stationary point for a single response or uses desirability functions to weight multiple responses, making it difficult to directly obtain a Pareto-optimal solution that simultaneously maximizes yield and BET while minimizing crystallite size. For such nonlinear multi-objective problems, machine-learning models (e.g., SVR, RF, XGBoost, and BP-ANN) combined with genetic-algorithm-based global search can often provide a richer set of trade-off solutions while maintaining low RMSE. Accordingly, RSM is well suited as an interpretable local surrogate and a tool for significance testing, whereas ML + GA is more suitable for strongly nonlinear fitting and multi-objective global optimization. Their integration can improve optimization quality while preserving statistical interpretability [38].

### 3.4 Prediction of key responses using different machine-learning models

**Fig. 15:**
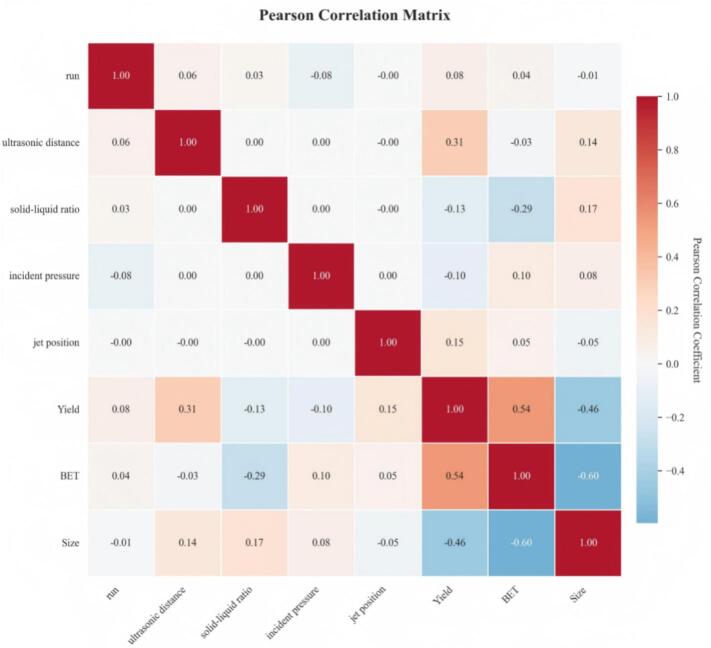
Pearson correlation heatmap.

As shown in the Pearson correlation heatmap in Fig. 15, the overall linear correlations between the four operating variables (ultrasonic distance, solid–liquid ratio, incident pressure, and jet position) and the three outputs (Yield, BET, and Size) are relatively weak (|R| < 0.35), indicating pronounced nonlinear coupling in the ultrasonic–jet carbonation system. Specifically, ultrasonic distance exhibits a weak correlation with yield (R = 0.31), which may be associated with variations in cavitation intensity, acoustic attenuation, and local microscale turbulent behavior. However, its correlations with BET (R = -0.03) and crystallite size (R = 0.14) are negligible, suggesting that the influence of distance on microstructural regulation is not linearly responsive. The solid–liquid ratio shows a weak negative correlation with BET (R = -0.29), implying that higher solids loading may intensify the competition between nucleation and growth, thereby reducing the specific surface area. In addition, a pronounced negative correlation is observed between BET and crystallite size (R = -0.60), indicating that smaller crystallites correspond to larger specific surface area, consistent with the geometric scaling of nanoparticles. Overall, while the linear correlations between process variables and the three key responses are weak, coupling relationships are evident among the responses themselves (e.g., Yield–BET and BET–Size). This suggests that the effects of operating parameters on product performance cannot be interpreted as single-factor linear contributions; rather, they arise from complex nonlinear interactions among cavitation intensity, jet-induced turbulence, local mass-transfer rates, interfacial renewal, and nucleation–growth kinetics. Therefore, this system is well suited to machine-learning models capable of capturing high-order nonlinearities and variable coupling for prediction and optimization.

**Fig. 16:**
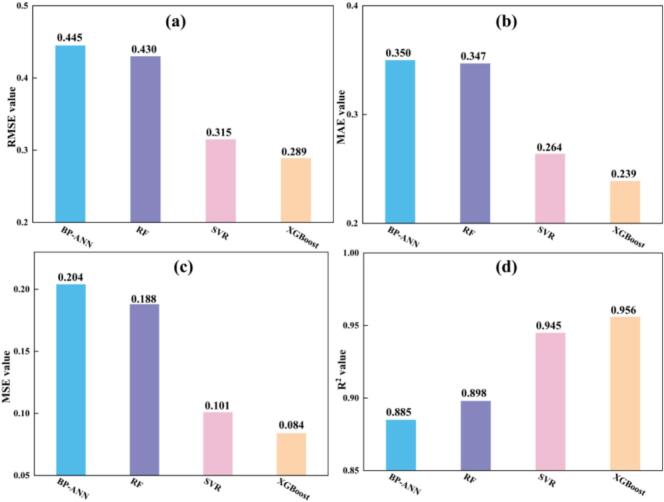
RMSE (a), MAE (b), MSE (c), and R^2^ (d) of different machine-learning models.

Based on the ANOVA results from the RSM analysis, the effects of these parameters on each response were nonlinear. Therefore, in the four ML models, four different parameters were used as the input variables, while three key quality indicators were taken as the output variables. As shown in Fig. 16, all four machine learning models achieved high prediction accuracy for reaction yield, specific surface area, and grain size under the multi-response joint modeling condition, although clear differences in predictive performance were observed. A comprehensive evaluation based on the overall error metrics of the three responses indicated that the XGBoost model exhibited the best overall performance, with MSE and MAE values of 0.084 and 0.239, respectively, which were significantly lower than those of BP-ANN (0.204 and 0.360) and RF (0.188 and 0.347). Its coefficient of determination (R^2^) also reached 0.956, the highest among the four models, indicating that XGBoost was better able to capture the strong nonlinear mechanisms in the ultrasonic jet carbonation process. SVR showed a relatively strong performance in terms of RMSE (0.315), but its MSE, MAE, and R^2^ were slightly inferior to those of XGBoost, placing it at a moderate level overall. BP-ANN and RF produced somewhat higher prediction errors, with R^2^ values of approximately 0.90, but they still significantly outperformed the traditional quadratic polynomial RSM in prediction accuracy.

**Fig. 17:**
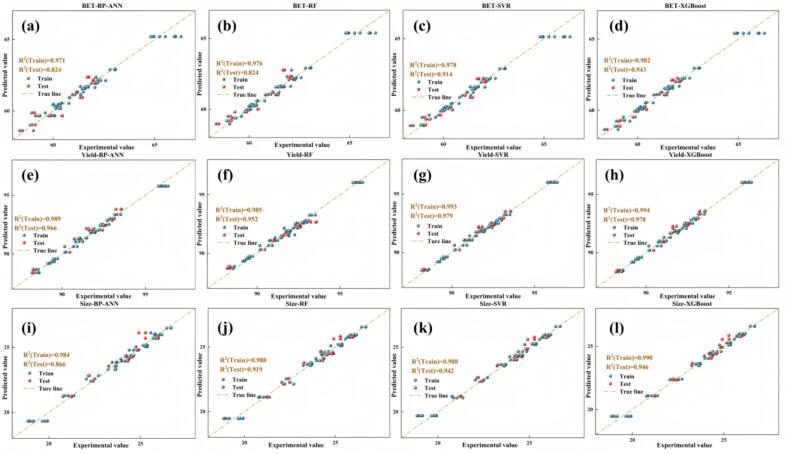
Comparison of the predictive performance of BP-ANN, RF, SVR, and XGBoost for the three quality attributes on the training and test sets.

The fitted scatter plots (Fig. 17a–l) provide a more intuitive assessment of each model’s capability to represent the three key quality attributes. By comparing the training and test sets, it can be observed that the predicted points from all four models are generally clustered close to the 45° identity line, with no evident systematic bias or extrapolation-induced distortion, indicating satisfactory generalization performance. Among them, XGBoost exhibits the most compact scatter clouds for all three responses, with R^2^ values for both the training and test sets approaching unity. In particular, in the regions of high yield, high BET, and small crystallite size, the predicted values almost overlap with the experimental measurements, demonstrating its superior ability to capture nonlinear variations arising from the synergistic effects of process parameters (e.g., interactions among temperature, pressure, and ultrasonic power) as well as the response behavior near the boundaries of the operating domain. The SVR model provides accurate fits for responses within the medium range; however, under extreme conditions (very high BET or very small crystallite size), slight underestimation and overestimation can be observed, suggesting that further optimization of kernel parameters and the penalty factor may be beneficial. RF and BP-ANN display typical “piecewise” or “smooth-interpolation” behavior: while they reproduce the overall experimental trends reasonably well, their scatter points deviate slightly from the identity line in data-sparse regions (e.g., under extreme operating conditions), indicating a relatively weaker capability to resolve localized complex kinetics compared with XGBoost.

Overall, all four machine-learning models can effectively predict the multiple quality attributes of nano-ZnO produced via ultrasonic–jet intensified carbonation. However, considering the aggregated error metrics and the fitting quality on both the training and test sets, XGBoost exhibits the highest accuracy for simultaneous multi-response prediction. Its superiority can be primarily attributed to the gradient-boosting strategy, which iteratively corrects residuals and is less prone than a single BP-ANN to becoming trapped in local optima. Moreover, for the present dataset—characterized by a low feature dimension (four variables) but strong nonlinearity and a relatively small sample size (tabular data)—tree-based XGBoost can naturally capture stepwise behaviors and feature interactions without requiring large datasets to tune network weights, as is typically the case for neural networks. In addition, the built-in regularization terms in XGBoost (L1/L2 regularization) effectively mitigate overfitting under limited-sample conditions, resulting in stronger generalization robustness than RF and SVR. Therefore, XGBoost was selected as the surrogate model for subsequent GA-based global optimization.

### 3.5 Genetic algorithm-based optimization of process parameters

**Fig. 18:**
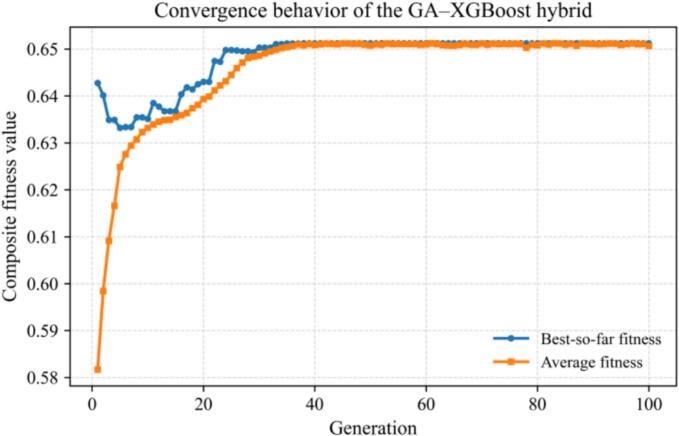
Iterative convergence curve of the GA–XGBoost hybrid optimization framework.

**Fig. 19:**
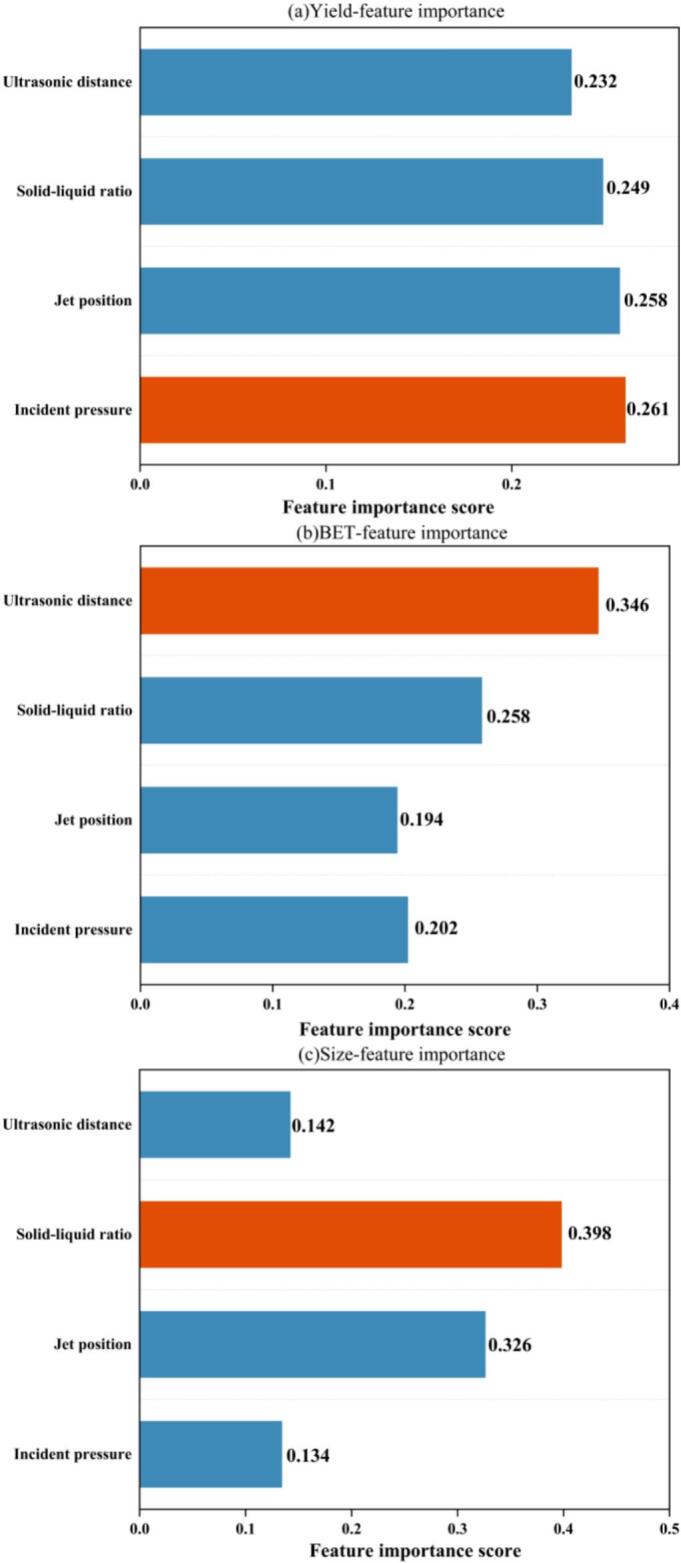
Comparison of feature importance of the GA–XGBoost model for predicting yield, BET, and crystallite size.

To achieve synergistic multi-response optimization beyond single-model performance evaluation, a GA–XGBoost hybrid intelligent optimization framework was developed. In this framework, XGBoost serves as a high-accuracy surrogate model for multi-response prediction, while the genetic algorithm (GA) performs global optimization over a continuous decision-variable space to simultaneously maximize reaction yield (Yield) and BET specific surface area (BET) and minimize crystallite size (Size). Fig. 18, Fig. 19(a–c) illustrate the optimization procedure and the contribution patterns of the responses, and Table 2 summarizes the optimal parameter set together with experimental validation. Fig. 18 presents the iterative convergence curve of GA guided by the XGBoost surrogate model. Parameter optimization was completed within 100 generations, with the objective-function value reaching 0.652. During generations 0 to 15, the best fitness rapidly increased from 0.58 to 0.645, while the mean fitness exhibited a steady upward trend. This indicates that GA effectively explored a broad parameter space in the early stage, and the XGBoost surrogate provided stable and reliable fitness evaluations, enabling high-quality individuals to be selected efficiently with good convergence behavior and without oscillation or degeneration. During generations 15 to 35, the fitness improvement became markedly slower, and the best value approached 0.652; the mean fitness nearly overlapped with the best fitness, suggesting that the population had entered a “high-quality solution cluster” with an overall stable solution quality. At this stage, mutation became the primary driver for fine adjustments, and GA conducted refined local searches to further improve the trade-off among the responses. After generation 35, the two fitness curves essentially coincided and no further improvement was observed, indicating convergence of the optimization. This also suggests a smooth surrogate-model response landscape with negligible multimodality, and a stable optimization process without apparent signs of overfitting.

**Table 2:** Comparison between predicted and experimental values.

Parameters	Predicted value	Data range	Experiment value
Reaction yield(％)	94.028	88.190 to 96.360	94.923 ± 0.285
BET(m^2^ g^−1^)	60.957	58.350 to 66.290	62.122 ± 0.535
Crystallite size(nm)	21.023	18.860 to 26.680	18.886 ± 0.595

To elucidate the specific effects of different process parameters on each quality attribute, feature importance for the three responses was extracted from the trained XGBoost model (Fig. 19a–c). Unlike Section 3.2, which focuses on “model accuracy,” the present analysis aims to clarify the dominant physical factors governing each response, thereby providing process- and mechanism-oriented insights. For reaction yield, the feature-importance values are relatively evenly distributed, with no single variable showing overwhelming dominance. This indicates that yield is a global response jointly influenced by multiple factors, including solid–liquid ratio, incident pressure, different jet positions, and ultrasonic distance, reflecting a clear “multifactor coupled regulation” behavior. This also explains why multi-objective integration is required in the GA-based optimization. In contrast, BET exhibits a pronounced hierarchical pattern in feature importance. Ultrasonic distance is the dominant factor (0.346), whereas the contributions of the other three variables are comparatively minor. This observation is highly consistent with the single-factor results (Fig. 9), which showed that ultrasonic distance governs the cavitation-field intensity. Specifically, BET formation is strongly dependent on the stripping/erosion of particle surfaces induced by cavitation microjets, and ultrasonic distance directly determines the efficiency of cavitation-energy delivery. This implies that BET is primarily controlled by cavitation intensity and its spatial distribution: an appropriate ultrasonic distance enhances the effectiveness of shock waves and microjets generated by bubble collapse for surface erosion and pore formation. The remaining process variables mainly modulate BET at a secondary level. Accordingly, BET can be regarded as a cavitation-dominated response, highly sensitive to local energy transfer and microstructural reconstruction. For crystallite size, the feature-importance distribution is highly concentrated, with solid–liquid ratio being the most influential factor (0.398), far exceeding the others, followed by incident pressure. This suggests that crystallite size is largely governed by the macroscopic concentration of the system and the probability of particle–particle interactions. A higher solid–liquid ratio increases the frequency of particle interactions, which favors secondary breakage and homogenization. Different jet positions affect the location of high-velocity turbulent zones and can therefore enhance fragmentation. Although ultrasonic distance and incident pressure also influence crystallite size, their roles are mainly associated with cavitation-driven “energy supplementation” rather than being the decisive factors. Consequently, crystallite size represents a typical mixing-kinetics-controlled response, driven by a dual mechanism combining “localized high-energy shear” and “bulk concentration effects.”

Based on the GA–XGBoost framework, the solution that maximized the comprehensive fitness function yielded the following optimal operating conditions for the ultrasonic–jet coupled carbonation process: ultrasonic transducer axial distance (L_9_ = 60.26) mm, solid–liquid ratio (= 5.72:100), incident pressure (= 0.756) MPa, and jet position (jet outlet height) (= 338.41) mm. Notably, both the solid–liquid ratio (5.72:100) and the incident pressure (0.756 MPa) are close to the upper bounds of the experimentally controllable range, indicating that the model tends to enhance reaction yield and BET by intensifying cavitation strength and mass-transfer efficiency. Moreover, the jet position was optimized to approximately 340 mm, near the maximum permitted by the reactor geometry, suggesting that stronger turbulent impingement and a higher density of cavitation-collapse events play a positive role in promoting uniform nucleation of ZnO-related nuclei.

To validate the extrapolation capability of the model and the reliability of the obtained optimum, additional experiments were conducted under the optimal conditions (reaction time 90 min, reaction temperature 80℃, ultrasonic power 700 W, ultrasonic transducer axial distance 60.26 mm, solid–liquid ratio 5.72:100, incident pressure 0.756 MPa, and jet position 338.41 mm). The predicted values were then compared with the experimentally measured results (Table 2). The three key performance indicators show high consistency. For reaction yield, the predicted value (94.028%) deviated by less than 1% from the experimental result (94.92 ± 0.29%). The predicted BET specific surface area (60.957 m^2^ g^−1^) differed from the measured value (62.12 ± 0.54 m^2^ g^−1^) by less than 2 m^2^ g^−1^. The predicted crystallite size was 21.023 nm, slightly higher than the measured value (18.89 ± 0.60 nm), but still on the order of ∼ 20 nm and following the correct optimization trend. Overall, the GA–XGBoost intelligent optimization framework can stably provide physically reasonable, experimentally reproducible optimal conditions with significantly improved performance under multi-factor coupling and multi-objective trade-offs, thereby offering a reliable mathematical basis and experimental evidence for subsequent process scale-up and industrial-level optimization.

### 3.6 Comparative analysis of products obtained under the optimal conditions

To quantify the specific contribution of the ultrasonic effect, comparative experiments were conducted using a jet-only mode (with the ultrasonic generator switched off) while keeping the other operating parameters at their optimized values (e.g., T = 80℃, P = 0.756 MPa, etc.).In parallel, calorimetric measurements were carried out for both jet-only and ultrasonic–jet operation under the same circulation configuration. During calorimetric testing, both the heating and cooling systems were switched off so that the measured temperature rise originated only from hydraulic recirculation and ultrasonic input. The 0 to 7 min interval was selected for linear fitting because it provided the best approximation of the initial linear temperature-rise region before the onset of thermal plateauing. Linear fitting yielded T = 21.592 + 0.1631 t (R^2^ = 0.981) for jet-only operation and T = 22.000 + 0.4429 t (R^2^ = 0.992) for ultrasonic–jet operation, where T is in °C and t is in min. Accordingly, the heating rates were determined to be 0.00272 and 0.00738 K s^−1^, respectively. Using 10 L of water for calorimetric determination, the corresponding thermal powers were calculated as 113.62 W for jet-only operation and 308.52 W for ultrasonic–jet operation, yielding a net ultrasonic calorimetric power of 194.90 W. These results confirm that ultrasonic coupling introduced a measurable additional energy input into the circulating liquid phase beyond the hydraulic baseline of the jet-only system. Table 3 summarizes both the calorimetric data and the key product-performance metrics of the ultrasonic–jet reactor and the conventional jet reactor for nano-ZnO production. The differences in all three indicators are far greater than the error range associated with experimental uncertainty, demonstrating that the intensification effect introduced by ultrasonic coupling is statistically significant.

**Table 3:** Comparison of calorimetric and product-performance parameters between ultrasonic–jet and conventional jet processes.

Parameters	Ultrasonic jet	Jet
Linear fitting equation	*T* = 22.000 + 0.4429*t*	*T* = 21.592 + 0.1631*t*
R^2^	0.992	0.981
Heating rate, *dT*/*dt* (K s^−1^)	0.00738	0.00272
Calorimetric power (W)	308.52	113.62
Net ultrasonic calorimetric power (W)	194.90	–
Reaction yield(％)	94.923 ± 0.285	81.231 ± 0.638
BET(m^2^ g^−1^)	62.122 ± 0.535	48.334 ± 0.279
Crystallite size(nm)	18.886 ± 0.595	34.661 ± 0.446

**Fig. 20:**
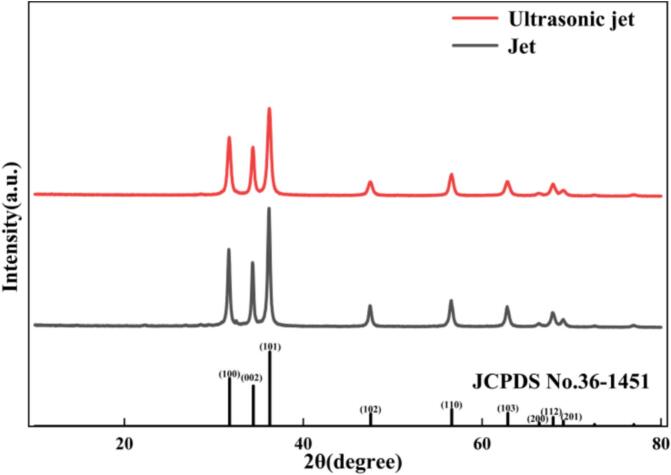
Comparison of XRD patterns of products prepared under ultrasonic–jet and conventional jet conditions.

Fig. 20 clearly illustrates the XRD phase characteristics of the products prepared under ultrasonic jet and conventional jet conditions. Qualitative analysis shows that all diffraction peak positions in both patterns are in excellent agreement with the standard card of hexagonal wurtzite ZnO (JCPDS No. 36-1451), with the characteristic reflections corresponding to the (100), (002), and (101) crystal planes, among others. No impurity peaks were detected, confirming that both processes are capable of producing high-purity ZnO crystals. However, a comparison of the peak profiles reveals a pronounced difference: relative to the conventional jet sample, the ultrasonic jet sample exhibits evident peak broadening, particularly for the three major reflections located at 31.7°, 34.4°, and 36.2°, as indicated by the increased full width at half maximum (FWHM). According to X-ray diffraction theory and the Scherrer equation, (D = Kλ/(βcosθ)), such peak broadening is directly associated with a reduction in crystallite size. This microstructural variation is fully consistent with the calculated results: the average crystallite size of the conventional jet sample is approximately 34.66 nm, corresponding to relatively sharp diffraction peaks, whereas that of the ultrasonic jet sample is significantly refined to 18.89 nm, leading to the observed peak broadening. This correlation between peak broadening and grain refinement provides strong evidence for the intensification mechanism induced by ultrasonic cavitation. Specifically, the extremely high nucleation density and intense micro-turbulent shear field introduced by ultrasound effectively disrupt the natural crystal growth equilibrium, thereby suppressing excessive grain growth and agglomeration and stabilizing the crystallite size at below 20 nm.

In terms of reaction yield, the ultrasonic–jet condition achieved 94.923 ± 0.285%, whereas the conventional jet-only mode reached only 81.231 ± 0.638%. This corresponds to an approximate 16.9% increase in yield for the ultrasonic–jet reactor relative to the conventional jet reactor. The BET specific surface area increased from 48.334 ± 0.279 m^2^ g^−1^ under jet-only operation to 62.122 ± 0.535 m^2^ g^−1^ under ultrasonic–jet operation, representing an improvement of ∼28.5%. Regarding crystallite size, the ultrasonic–jet process produced a crystallite size of 18.886 ± 0.595 nm, which is substantially smaller than that obtained under jet-only conditions (34.661 ± 0.446 nm).Overall, the ultrasonic–jet coupling reduced crystallite size by approximately 45.5%. These results demonstrate that acoustically induced cavitation and intensified micromixing significantly promote the carbonation process. Under the synergistic action of ultrasound and jetting, the resulting ZnO exhibits a more developed porous structure and a larger specific interfacial area, while enhanced uniform nucleation and suppression of crystal growth and agglomeration lead to nano-ZnO with smaller crystallites and a narrower size distribution.

**Fig. 21:**
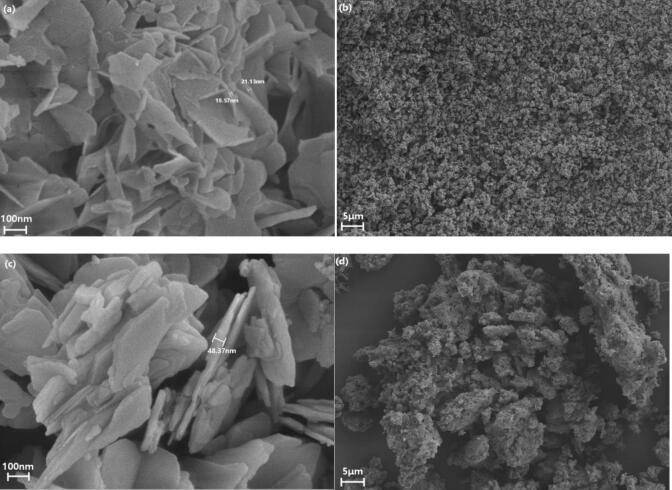
SEM comparison of products prepared under ultrasonic–jet and conventional jet conditions.

The micro-morphologies of nano-ZnO produced by the ultrasonic–jet reactor and the conventional jet reactor were examined by scanning electron microscopy (SEM), and the results are shown in Fig. 21. Fig. 21(a) and 21(b) present high- and low-magnification SEM images of the nano-ZnO obtained under ultrasonic–jet conditions, while Fig. 21(c) and 21(d) show the corresponding images for the jet-only sample.The jet-only sample exhibits relatively dense agglomerates composed of irregular flake-like or blocky particles stacked in a disordered manner. The surfaces are rough and the pore structure is heterogeneous, with pronounced secondary agglomeration in some regions. These features suggest that under weaker shear and mass-transfer conditions, particle growth is largely diffusion-controlled; after nucleation, crystallites readily coalesce and densify, resulting in compact aggregates. In contrast, the ultrasonic–jet sample displays a more regular, well-dispersed lamellar/flaky morphology. Individual nanosheets show clear outlines with sharp edges, a more uniform size distribution, and a certain degree of oriented stacking. The overall architecture is loose and porous, forming a three-dimensional network reminiscent of “flower-like” or “stacked-sheet” structures. This markedly improved morphology can be attributed to the transient high-energy microdomains and intense turbulent shear introduced by ultrasonic cavitation, which enhance mixing uniformity while promoting high-density nucleation and controlled crystal growth. Moreover, cavitation-induced stripping and re-dispersion at the early growth stage suppress disordered agglomeration and excessive coarsening. In addition, periodic fragmentation and reorganization driven by ultrasound further activate particle surfaces, favoring preferential growth of lamellar crystallites along specific crystallographic directions and leading to a more open porous structure. Such a microstructure—characterized by high specific surface area, low agglomeration, and improved structural order—suggests enhanced interfacial reactivity and mass-transfer performance in applications such as catalysis, adsorption, and sensing, highlighting the advantages of the ultrasonic–jet reactor for microstructural regulation and material-property optimization.

**Fig. 22:**
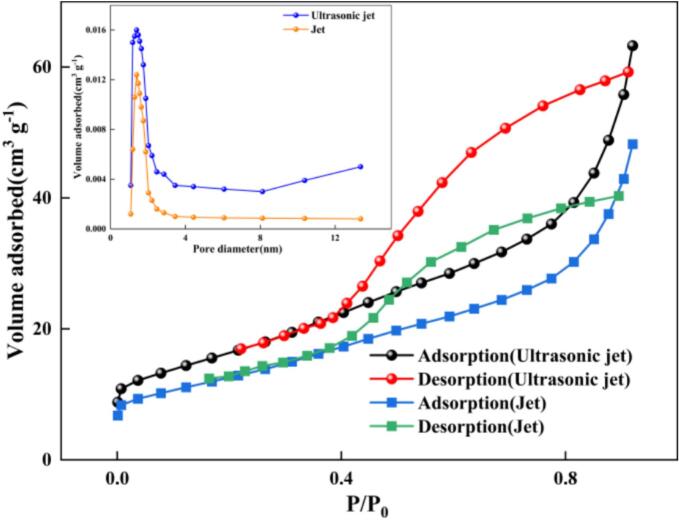
Comparison of N_2_ adsorption–desorption isotherms for samples prepared under ultrasonic–jet and conventional jet conditions.

Nitrogen adsorption–desorption measurements were performed using a fully automated surface area analyzer, and the results are shown in Fig. 22. The adsorption–desorption isotherms and the corresponding pore-size distribution curves reveal pronounced differences in pore-structure characteristics and specific surface area between the samples prepared by the ultrasonic–jet reactor and the conventional jet reactor. Both samples exhibit typical type-IV isotherms with evident hysteresis loops, indicating predominantly mesoporous structures. However, the ultrasonic–jet sample shows a steeper adsorption uptake and a higher adsorbed volume in the medium-to-high relative pressure region ((P/P_0_ > 0.4)), implying a higher specific surface area and a more developed pore network. In addition, its hysteresis loop is wider and more regular in shape, suggesting a more open pore architecture with stronger pore connectivity, which is favorable for gas diffusion and transport. By comparison, the jet-only sample displays a generally lower adsorption capacity and a more gradual uptake, indicating a relatively denser pore structure with fewer effective pores. The pore-size distribution further confirms that the ultrasonic–jet sample has a more concentrated distribution, mainly within a smaller and more uniform mesopore range. This suggests that ultrasonic cavitation and the intensified shear field effectively suppress disordered particle packing and pore collapse, thereby promoting the formation of more regular pore structures. In contrast, the jet-only sample exhibits a broader pore-size distribution, reflecting higher heterogeneity in pore structure and a greater degree of agglomeration. Overall, by enhancing micromixing, accelerating nucleation, and regulating crystal growth, the ultrasonic–jet reactor markedly optimizes the pore-structure features of the product, yielding higher specific surface area, a more favorable mesopore distribution, and improved pore connectivity. These structural advantages provide a solid basis for enhanced interfacial reactivity and mass-transfer efficiency in applications such as catalysis, adsorption, and other functional uses.

**Fig. 23:**
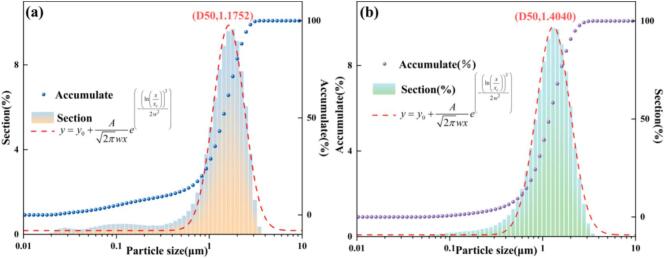
Comparison of particle-size distributions of samples prepared under ultrasonic–jet and conventional jet conditions.

Fig. 23 presents the particle-size distribution results of nano-ZnO samples prepared using the ultrasonic–jet reactor and the conventional jet reactor, indicating an overall good monodispersity in the particle-size characteristics. The particle-size distribution curves reveal fundamental differences in particle scale and distribution uniformity between the two samples. Both samples exhibit unimodal distributions, suggesting that the dominant nucleation–growth mechanism is broadly similar. However, the ultrasonic–jet sample shows a sharper peak with a noticeably narrower full width at half maximum, and its D50 is concentrated in a smaller particle-size range, demonstrating significant size refinement and higher size uniformity. This behavior reflects that the high-frequency microjets and transient high-shear fields induced by ultrasonic cavitation effectively enhance precursor mixing and nucleation density, shifting the system from a “few-nuclei/rapid-growth” regime to a “many-nuclei/controlled-growth” regime, thereby suppressing excessive coarsening and agglomeration. In contrast, the jet-only sample exhibits a broader particle-size distribution with a pronounced tail, indicating the presence of a certain fraction of coarse particles. This suggests that under weaker perturbation, particle growth is more diffusion-controlled and is therefore more prone to secondary aggregation and non-uniform coarsening. Combined with the SEM and BET results discussed above, it can be inferred that ultrasonic–jet coupling not only refines the particle scale but also improves the concentration and stability of the particle-size distribution. Such “small and uniform” particle characteristics are beneficial for increasing specific surface area, enhancing interfacial activity, and improving process controllability in subsequent applications, further highlighting the advantages of the ultrasonic–jet reactor in microstructural regulation and overall performance enhancement.

### 3.7 Comparison with a published hydrodynamic cavitation-enhanced route

To further position the proposed method within the current literature, a comparison was made with a representative published intensified route for nano-ZnO synthesis, namely the hydrodynamic cavitation-enhanced carbonization method reported by Guo et al. As summarized in Table 4, the present ultrasonic–jet coupled route achieved a reaction yield of 94.923 ± 0.285%, a BET specific surface area of 62.122 ± 0.535 m^2^ g^−1^, and an XRD-based size metric of 18.886 ± 0.595 nm under optimized conditions of 90 min and 80°C. By comparison, the published hydrodynamic cavitation route required 120 min at the same temperature and produced a carbonization rate of 93.937%, a BET specific surface area of 62.377 m^2^ g^−1^, and an XRD-based size metric of 28.679 nm. The comparison indicates that the proposed method reduces the reaction time while maintaining a comparable BET level and a slightly higher conversion performance. A more notable difference is observed in the product size metric, with the present route yielding a smaller value than the published hydrodynamic cavitation route. This difference may be attributed to the synergistic effect of jet-induced shear and ultrasonic cavitation. Nevertheless, the present route also requires additional ultrasonic equipment and energy input, resulting in higher system complexity. Therefore, the hydrodynamic cavitation route remains attractive from the viewpoint of process simplicity, whereas the proposed ultrasonic–jet coupled route provides a more favorable balance among processing time, yield, and product refinement.

**Table 4:** Comparison between the present method and a published hydrodynamic cavitation route for nano-ZnO synthesis.

Method	Ultrasonic–jet coupled carbonation	Hydrodynamic cavitation-enhanced carbonization (Guo et al.[39])
Time(min)	90	120
Temperature(℃)	80	80
Yield/carbonization rate(％)	94.923 ± 0.285	93.937
BET(m^2^ g^−1^)	62.122 ± 0.535	62.377
Crystallite / grain size (nm)	18.886 ± 0.595	28.679
Main advantages	Shorter processing time; finer crystallite size	relatively simple cavitation-based intensification
Main limitations	Additional ultrasonic equipment; higher system complexity	Longer treatment time; larger grain size; weaker microstructure refinement

## 4 Conclusion

This study systematically investigates the process mechanism, parameter optimization, and intelligent prediction of nano-ZnO synthesis via ultrasonic–jet reactor–intensified carbonation. By integrating experiments, response surface methodology (RSM), and multiple machine-learning models coupled with genetic-algorithm-based optimization, the following main conclusions are drawn:

1. Compared with the conventional jet reactor, the ultrasonic–jet reactor under the optimal conditions increased the reaction yield from 81.23% to 94.92% and the BET specific surface area from 48.33 to 62.12 m^2^ g^−1^, while reducing the crystallite size from 34.66 to 18.89 nm, corresponding to improvements of approximately 16.9%, 28.5%, and 45.5%, respectively. The high-velocity shear, turbulence, and internal recirculation induced by the jet markedly enhanced the CO_2_ mass-transfer rate and accelerated the fragmentation and renewal of the carbonation layer on the ZnO surface. In parallel, ultrasonic cavitation generated microjets, high-energy shock waves, and transient localized high-temperature/high-pressure microdomains, which further weakened the self-passivating carbonation shell and promoted the continuous progression of dissolution–nucleation–growth processes. The superposition of these two effects substantially intensified the carbonation kinetics of ZnO, shifting the reaction from diffusion-controlled behavior to a nucleation-dominated regime, thereby achieving comprehensive improvements in key quality attributes, including reaction yield, specific surface area, and crystallite size.Calorimetric analysis further confirmed that ultrasonic coupling introduced a measurable net acoustic power of 194.90 W, thereby providing a quantitative energetic basis for the observed process intensification.
2. The BBD-based response surface models achieved coefficients of determination R^2^ of 0.8764, 0.8648, and 0.8489 for the three quality attributes, confirming the significant influence of process parameters on the responses. However, due to the inherent limitations of quadratic polynomial formulations, RSM shows deficiencies in multi-objective synergistic optimization and in capturing strongly nonlinear response behavior.
3. Among the four models (BP-ANN, SVR, RF, and XGBoost), XGBoost delivered the best multi-response prediction performance, achieving an overall R^2^ of 0.956 with MSE and MAE of 0.084 and 0.239, respectively. It can more accurately capture the complex nonlinear behavior of the ultrasonic–jet coupled carbonation process, showing particularly high stability in the regions of high BET and small crystallite size; therefore, it was selected as the surrogate model for the GA optimization stage. Using an equal-weight multi-objective fitness function, the optimal operating conditions were obtained as: t = 90 min, T = 80℃, ultrasonic power = 700 W, ultrasonic transducer axial distance L_9_ = 60.26 mm, solid–liquid ratio = 5.72:100, incident pressure = 0.756 MPa, and jet outlet height = 338.41 mm. Experimental validation showed excellent agreement with the predicted values, with all deviations below 5%, confirming the reliability and practicality of the GA–XGBoost framework for coupled multi-parameter optimization.
4. SEM observations reveal that the products obtained via ultrasonic–jet processing exhibit a well-defined porous network composed of regularly stacked lamellar units, with a homogeneous particle distribution and a low degree of agglomeration. BET surface area measurements combined with N_2_ adsorption–desorption analysis indicate a more developed mesoporous architecture and a narrower, more concentrated pore-size distribution. Moreover, crystallite-size distribution results further confirm an improved monodispersity, which is expected to enhance interfacial reactivity and thereby benefit catalytic, adsorption, and other functional applications.

## CRediT authorship contribution statement

**Jinyuan Guo:** Writing – review & editing, Writing – original draft, Visualization, Validation, Methodology, Investigation, Formal analysis, Data curation, Conceptualization. **Honglei Yu:** Funding acquisition. **Dexi Wang:** Funding acquisition. **Lin Fan:** Funding acquisition.

## Declaration of competing interest

The authors declare that they have no known competing financial interests or personal relationships that could have appeared to influence the work reported in this paper.

## Appendix A Supplementary data

Supplementary Data 1 Table S1 presents the complete Box–Behnken experimental design matrix and corresponding response values, while Table S2 summarizes the ANOVA results of the quadratic response-surface models for reaction yield, BET specific surface area, and crystallite size.

## References

[1] Sabuad A., Khaokong C., Kongseng P., Chantarak S. (2024). Superabsorbent ZnO/rubber-based hydrogel composite for removal and photocatalytic degradation of methylene blue. Int. J. Biol. Macromol..

[2] Pollard Z.A., Karod M., Schmitz A., Pian B., Barstow B., Goldfarb J.L. (1996). ZnO Precursor’s ability to catalyze formation of reactive oxygen species to degrade aqueous organic pollutants. Chem. Eng. J. (lausanne Switzerland).

[3] Dogan C., Martini S., Retschitzegger S., Çetin B. (2023). The effect of the presence of water on sulfur removal capacity during H2S removal from syngas using ZnO adsorbent. Environ. Technol..

[4] Zheng S., Chen W., Shi C., Han J. (2024). Thermostable ZnO/Ag@Si nanohybrid material for extraordinary antibacterial activity polyester fibers. Polym. Eng. Sci..

[5] Lin Y., Lin Y., Wu J., Zhang X., Fang B. (2017). Fabrication of ZnO/SnO2 hierarchical structures as the composite photoanodes for efficient CdS/CdSe co-sensitized solar cells. Appl. Phys. A Mater. Sci. Process..

[6] Savalia D., Anadani P., Das Lala S. (2026). Effect of surfactant type on the stability and thermophysical performance of ZnO–ethylene glycol nano fluids. Discov. Mech. Eng..

[7] Hayder A., Kuila S.K., Mazhkoo S., Santos R.M., Dutta A. (2025). Development of advanced activated biocarbon from corn distiller soluble via two-step carbonization: investigating the synergistic effects of ZnO and K toward enhanced CO2 capture. ACS Sustain. Chem. Eng..

[8] Zheng W., Liu Q., Zeng X., Li M., Liu Y., Yang D., Li X., Weng W., Zhang Y. (2025). Sodium alginate/bacterial cellulose nanofibers films enhanced by ZnO nanoparticles and aqueous phase from hydrothermal carbonization for functional food packaging. Int. J. Biol. Macromol..

[9] Cao S., Wu Z., Sun Q., Zhang W., Beysen S., Wang S., Shaymurat T., Zhang M., Duan H. (2021). Gas sensing properties of cotton-based carbon fibers and ZnO/carbon fibers regulated by changing carbonization temperatures. Sens. Actuators B.

[10] Murphy P.J., Alimohammadi S., Shaughnessy S.M.O. (2025). An experimental investigation of the effect of the velocity ratio on the flow and wall heat transfer characteristics of a wall-bounded dual jet. Therm. Sci. Eng. Prog..

[11] Lin F., Li M. (2026). Bubble entrainment in turbulent jets leaping from liquid surface. Int. J. Heat Fluid Flow.

[12] Riaz S., Aaltonen J., Koskinen K. (2025). Performance mapping of modular annular jet pumps for slurry transport—A mixture model approach. Chemical Engineering Journal Advances.

[13] Zuo M., Yu H., Wang D., Fan L. (2024). Jet cavitation-enhanced hydration method for the preparation of magnesium hydroxide. Chem. Eng. Process..

[14] Guo J., Yu H., Wang D., Chen G., Fan L. (2025). Experimental study, mechanism, and process optimization of hydrodynamic cavitation-enhanced green synthesis of nano ZnO using Eupatorium Adenophorum. Chem. Eng. Process..

[15] Yang H., Wang D., Yang Y., Jiang J., Yu H., Li S., Guo J. (2025). Multiphase flow dynamics analysis in jet cavitation nozzles: a novel approach for optimizing oily sludge treatment efficiency. Chin. J. Phys. (taipei).

[16] Stephens D.S., Troia A., Cravotto G., Martina K., Mettin R. (2025). Ultrasound combined with microwave irradiation: cavitation regimes and acoustic emissions. Ultrason. Sonochem..

[17] Xiong G., Ren Y., Fang Z., Jia X., Sun K., Guo B., Huang Q., Wang C., Zhou S. (2024). Understanding the cavitation effect of power ultrasound in cement paste. Constr. Build. Mater..

[18] Li G., Zhao Y., Li J., Xiao Y. (2023). Evolution behavior of cavitation bubble in pure Sn liquid medium with narrow gap under low-amplitude ultrasound. Ultrason. Sonochem..

[19] Ručigaj A., Connell J.G., Dular M., Genorio B. (2022). Influence of the ultrasound cavitation intensity on reduced graphene oxide functionalization. Ultrason. Sonochem..

[20] Parvizian F., Rahimi M., Faryadi M. (2011). Macro- and micromixing in a novel sonochemical reactor using high frequency ultrasound. Chem. Eng. Process..

[21] Liu B., Duan L., Cai S., Ren Q., Li J., Wang Y., Zeng Y. (2025). A clean and efficient route for extraction of vanadium from vanadium slag by electro-oxidation combined with ultrasound cavitation (vol 102, 106735, 2024). Ultrason. Sonochem..

[22] Chen L., Zeng H., Guo Y., Yang X., Chen B. (2022). A comparative analysis of micro-mixing process in a confined impinging jet reactor with/without applying ultrasound. Chem. Eng. Process..

[23] Lu J., Guo Y., Dong B., Yang X., Li J. (2024). Turbulence-assisted shear controllable synthesis of silicon oxide micro/nano particles using a counter axial-swirling impinging jet flow reactor. Colloids Surf A Physicochem Eng Asp.

[24] Kadhum A.M., Waheeb A.S., Mallah S.H., Awad M.A., Hasan D.M., Kyhoiesh H.A.K., Abd Elsalam H.E., El Azab I.H. (2026). Integrating computational chemistry and machine learning for the stability prediction of transition metal ternary compounds. Mater. Chem. Phys..

[25] Adcock K., Arulchelvan E., Shields N., Vanneste S. (2026). Towards precision medicine for otology and neurotology: machine learning applications and challenges. Hearing Res.

[26] Suppakul R., Jitchaijaroen W., Keawsawasvong S., Intui S., Inazumi S. (2025). Undrained uplift capacity prediction of open-caisson anchors in anisotropic clays using XGBoost integrated with mutation-based genetic algorithms. Artif. Intell. Geosci..

[27] Chi M., Zeng X., Gao Y., Li W., Jiang H., Sun R. (2024). The erosion rate prediction for the elbow in shale gas gathering and transportation system: RSM and GA-BP-ANN modeling. Powder Technol..

[28] Zhang Y., Bao X., Zhu Y., Dai Z., Shen Q., Xue Y. (2024). Advances in machine learning screening of food bioactive compounds. Trends Food Sci. Tech..

[29] Hu B., Zhang C., Chu B., Gu P., Zhu B., Qian W., Chang X., Yu M., Zhang Y., Wang X. (2023). Unraveling the relationship between key aroma components and sensory properties of fragrant peanut oils based on flavoromics and machine learning. Food Chem.: X.

[30] Qiu Y., Zhou J., Khandelwal M., Yang H., Yang P., Li C. (2022). Performance evaluation of hybrid WOA-XGBoost GWO-XGBoost and BO-XGBoost models to predict blast-induced ground vibration. Eng. Comput.-Germany.

[31] Kumar A., Pathak A.K., Guria C. (2015). NPK-10:26:26 complex fertilizer assisted optimal cultivation of Dunaliella tertiolecta using response surface methodology and genetic algorithm. Bioresour. Technol..

[32] Behrens M.A., Franzén A., Carlert S., Skantze U., Lindfors L., Olsson U. (2025). On the Ostwald ripening of crystalline and amorphous nanoparticles. Soft Matter..

[33] Khavari M., Priyadarshi A., Morton J., Porfyrakis K., Pericleous K., Eskin D., Tzanakis I. (2023). Cavitation-induced shock wave behaviour in different liquids. Ultrason. Sonochem..

[34] Edsall C., Huynh L., Mustafa W., Hall T.L., Durmaz Y.Y., Vlaisavljevich E. (2024). Nanoparticle-mediated histotripsy using dual-frequency pulsing methods. Ultrasound Med. Biol..

[35] Jia B., Soyama H. (2024). Non-spherical cavitation bubbles: a review. Fluids.

[36] Maeda K., Maxwell A.D. (2021). Controlling the dynamics of cloud cavitation bubbles through acoustic feedback. Phys. Rev. Appl.

[37] Soyama H. (2021). Luminescence intensity of vortex cavitation in a Venturi tube changing with cavitation number. Ultrason. Sonochem..

[38] Zeng J., Luo J., Song Y., Jiang X., Cong P., Liu Y., Xue C., Xu J. (2025). Multi-quality attributes prediction and process parameter optimization of liquid-smoked rainbow trout by different machine learning models combined with genetic algorithm. Food Res. Int..

[39] Guo J., Yu H., Wang D., Chen G., Fan L., Yang H. (2024). Experimental study on preparation of nano ZnO by hydrodynamic cavitation-enhanced carbonization method and response surface optimization. Processes.

