# Supplementary material for: Cavitation-enhanced carbonation for nano-ZnO synthesis via an ultrasonic–jet coupled reactor: Machine learning prediction and multi-objective optimization using a genetic algorithm
Source: Ultrason Sonochem. 2026 Apr 17;129:107859. doi: 10.1016/j.ultsonch.2026.107859 (PMC13103568; doi:10.1016/j.ultsonch.2026.107859)
Supplement: Supplementary Data 1 — Table S1 presents the complete Box–Behnken experimental design matrix and corresponding response values, while Table S2 summarizes the ANOVA results of the quadratic response-surface models for reaction yield, BET specific surface area, and crystallite size. [file mmc1.docx]

Supplementary Materials: Table S1 provides the complete Box–Behnken experimental design matrix and the corresponding response values. Table S2 summarizes the ANOVA results of the quadratic response-surface models for reaction yield, BET specific surface area, and crystallite size.

Tab.S1 Box–Behnken experimental design matrix and corresponding response values.

| Run | A:A-Ultrasonic distance | B-Solid-liquid ratio | C-Incident pressure | D-Different jet positions | Reaction yield(％) | BET(m^2^ g^-1^) | Crystallite size(nm) |
| --- | --- | --- | --- | --- | --- | --- | --- |
| 1 | 40 | 0.05 | 0.7 | 350 | 90.96±0.32 | 59.87±0.18 | 22.43±0.43 |
| 2 | 60 | 0.04 | 0.7 | 300 | 93.02±0.56 | 58.94±0.05 | 24.51±0.52 |
| 3 | 50 | 0.05 | 0.6 | 250 | 91.02±0.33 | 61.74±0.09 | 25.33±0.69 |
| 4 | 40 | 0.05 | 0.8 | 300 | 88.19±0.43 | 61.33±0.08 | 21.26±0.28 |
| 5 | 50 | 0.05 | 0.8 | 350 | 93.23±0.25 | 61.68±0.19 | 24.80±0.64 |
| 6 | 50 | 0.04 | 0.7 | 250 | 89.27±0.46 | 62.58±0.06 | 23.72±0.76 |
| 7 | 40 | 0.05 | 0.6 | 300 | 92.62±0.53 | 60.94±0.12 | 22.16±0.55 |
| 8 | 50 | 0.05 | 0.7 | 300 | 95.87±0.40 | 64.89±0.05 | 19.89±0.39 |
| 9 | 60 | 0.05 | 0.8 | 300 | 91.97±0.28 | 61.52±0.07 | 22.52±0.53 |
| 10 | 50 | 0.04 | 0.8 | 300 | 89.49±0.33 | 63.07±0.16 | 25.77±0.36 |
| 11 | 60 | 0.06 | 0.7 | 300 | 91.57±0.67 | 59.12±0.31 | 25.94±0.71 |
| 12 | 50 | 0.06 | 0.8 | 300 | 92.57±0.22 | 61.15±0.22 | 26.66±0.67 |
| 13 | 40 | 0.06 | 0.7 | 300 | 88.34±0.25 | 58.49±0.09 | 24.18±0.30 |
| 14 | 40 | 0.05 | 0.7 | 250 | 88.57±0.17 | 62.29±0.11 | 23.68±0.56 |
| 15 | 50 | 0.05 | 0.7 | 300 | 96.46±0.28 | 64.72±0.33 | 20.62±0.35 |
| 16 | 50 | 0.04 | 0.7 | 350 | 91.94±0.09 | 62.15±0.24 | 24.62±0.18 |
| 17 | 50 | 0.06 | 0.6 | 300 | 90.22±0.38 | 59.28±0.26 | 25.73±0.29 |
| 18 | 50 | 0.05 | 0.8 | 250 | 90.68±0.51 | 59.98±0.21 | 25.84±0.56 |
| 19 | 50 | 0.05 | 0.6 | 350 | 92.52±0.55 | 62.04±0.15 | 24.31±0.48 |
| 20 | 50 | 0.06 | 0.7 | 350 | 89.36±0.52 | 60.43±0.34 | 25.82±0.54 |
| 21 | 60 | 0.05 | 0.6 | 300 | 91.48±0.76 | 60.37±0.19 | 21.37±0.70 |
| 22 | 60 | 0.05 | 0.7 | 250 | 93.16±0.41 | 60.01±0.05 | 24.64±0.67 |
| 23 | 50 | 0.06 | 0.7 | 250 | 91.83±0.39 | 60.43±0.13 | 25.28±0.61 |
| 24 | 40 | 0.04 | 0.7 | 300 | 92.76±0.42 | 60.09±0.08 | 23.66±0.74 |
| 25 | 60 | 0.05 | 0.7 | 350 | 92.18±0.68 | 61.73±0.27 | 24.38±0.59 |
| 26 | 50 | 0.04 | 0.6 | 300 | 92.44±0.05 | 61.42±0.14 | 25.26±0.86 |
| 27 | 50 | 0.05 | 0.7 | 300 | 96.13±0.36 | 65.96±0.09 | 19.17±0.37 |

Tab.S2 Analysis of variance (ANOVA) results for the quadratic response-surface models of reaction yield, BET specific surface area, and crystallite size

| Source | DF | Reaction yield | | | BET | | | Crystallite size | | |
| --- | --- | --- | --- | --- | --- | --- | --- | --- | --- | --- |
|  |  | Sum of Squares | F | P | Sum of Squares | F | P | Sum of Squares | F | P |
| Model | 14 | 108.71 | 6.07 | 0.0017 | 74.04 | 5.48 | 0.0027 | 88.54 | 4.82 | 0.0048 |
| A-Ultrasonic distance | 1 | 11.88 | 9.29 | 0.0101 | 0.1452 | 0.1506 | 0.7048 | 2.99 | 2.28 | 0.1572 |
| B-Solid-liquid ratio | 1 | 2.11 | 1.65 | 0.2233 | 7.29 | 7.56 | 0.0176 | 3.07 | 2.34 | 0.1522 |
| C-Incident pressure | 1 | 1.45 | 1.13 | 0.3079 | 0.7203 | 0.7471 | 0.4044 | 0.6030 | 0.4592 | 0.5109 |
| D-Different jet positions | 1 | 2.67 | 2.09 | 0.1740 | 0.0631 | 0.0654 | 0.8025 | 0.3781 | 0.2879 | 0.6014 |
| AB | 1 | 2.21 | 1.73 | 0.2136 | 0.7921 | 0.8215 | 0.3826 | 0.2070 | 0.1576 | 0.6983 |
| AC | 1 | 6.05 | 4.73 | 0.0503 | 0.1444 | 0.1498 | 0.7055 | 1.05 | 0.8000 | 0.3887 |
| AD | 1 | 2.84 | 2.22 | 0.1619 | 4.28 | 4.44 | 0.0567 | 0.2450 | 0.1866 | 0.6734 |
| BC | 1 | 7.02 | 5.49 | 0.0371 | 0.0121 | 0.0125 | 0.9127 | 0.0441 | 0.0336 | 0.8577 |
| BD | 1 | 6.60 | 5.17 | 0.0422 | 0.0462 | 0.0479 | 0.8304 | 0.0324 | 0.0247 | 0.8778 |
| CD | 1 | 0.2756 | 0.2156 | 0.6507 | 0.4900 | 0.5082 | 0.4895 | 0.0001 | 0.0001 | 0.9932 |
| A^2^ | 1 | 31.98 | 25.02 | 0.0003 | 43.87 | 45.50 | ＜0.0001 | 3.56 | 2.71 | 0.1257 |
| B^2^ | 1 | 38.88 | 30.42 | 0.0001 | 35.00 | 36.30 | ＜0.0001 | 60.54 | 46.10 | ＜0.0001 |
| C^2^ | 1 | 26.94 | 21.08 | 0.0006 | 14.35 | 14.89 | 0.0023 | 23.56 | 17.94 | 0.0012 |
| D^2^ | 1 | 32.31 | 25.28 | 0.0003 | 13.47 | 13.97 | 0.0028 | 35.55 | 27.07 | 0.0002 |
| Residual | 12 | 15.34 |  |  | 11.57 |  |  | 15.76 |  |  |
| Lack of Fit | 10 | 15.16 | 17.34 | 0.0557 | 10.67 | 2.36 | 0.3341 | 14.71 | 2.80 | 0.2919 |
| R^2^ | 0.8764 | | | | 0.8648 | | | 0.8489 | | |
| Adjusted R^2^ | 0.7321 | | | | 0.7072 | | | 0.6726 | | |
